# A computational model to design wide field-of-view optic nerve neuroprostheses

**DOI:** 10.1016/j.isci.2024.111321

**Published:** 2024-11-05

**Authors:** Simone Romeni, Daniela De Luca, Luca Pierantoni, Laura Toni, Gabriele Marino, Sara Moccia, Silvestro Micera

**Affiliations:** 1 Modular Implantable Neurotechnologies Laboratory, Università Vita-Salute San Raffaele & Scuola Superiore Sant’Anna, Milan, Italy; 2 Bertarelli Foundation Chair in Translational Neural Engineering, Center for Neuroprosthetics and Institute of Bioengineering, Ecole Polytechnique Federale de Lausanne, Lausanne, Switzerland; 3 The Biorobotics Institute and Department of Excellence in Robotics and AI, Scuola Superiore Sant’Anna, Pisa, Italy; 4 Brain Connectivity Laboratory, Department of Neuroscience and Neurorehabilitation, IRCCS San Raffaele Roma, Rome, Italy; 5 Department of Innovative Technologies in Medicine and Dentistry, Università degli Studi “G. d’Annunzio”, Chieti-Pescara, Italy

**Keywords:** Neuroscience, Computational bioinformatics

## Abstract

Retinal stimulation (RS) allows restoring vision in blind patients, but it covers only a narrow region of the visual field. Optic nerve stimulation (ONS) has the potential to produce visual perceptions spanning the whole visual field, but it produces very irregular phosphenes. We introduced a geometrical model converting retinal and optic nerve firing rates into visual perceptions and vice versa and a method to estimate the best perceptions elicitable through an electrode configuration. We then compared in silico ONS and RS through simulated prosthetic vision of static and dynamic visual scenes. Both simulations and SPV experiments showed that it might be possible to reconstruct natural visual scenes with ONS and RS, and that ONS wide field-of-view allows the perception of more detail in dynamic scenarios than RS. Our findings suggest that ONS could represent an interesting approach for vision restoration and that our model can be used to optimize it.

## Highlights

• Retina and optic nerve electrical stimulation have very different peculiar features
• We built an in-silico model to estimate the best perceptions from an electrode grid
• We compared retinal and optic nerve stimulation through psychophysical experiments
• Optic nerve stimulation allows to identify more objects in a dynamic scene

## Graphical abstract

**Figure:**
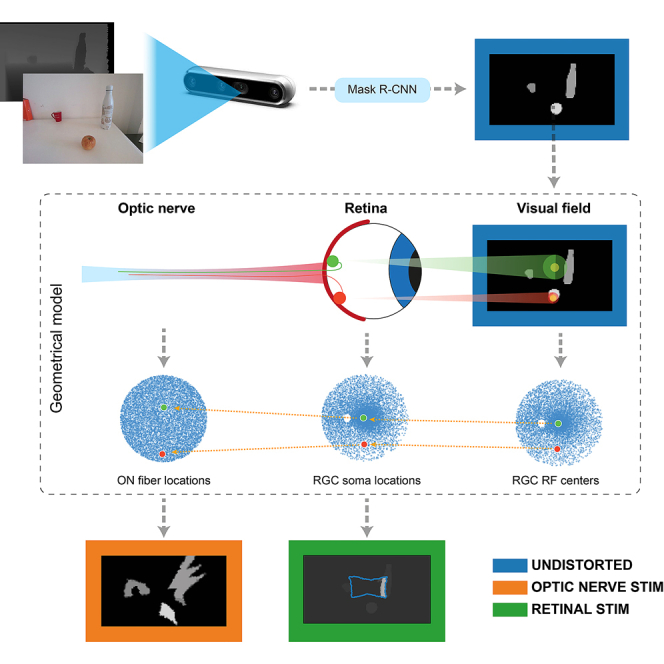

## Introduction

Many individuals with acquired blindness suffer from retinal tissue degeneration^1^ and could benefit from visual prostheses that electrically stimulate the retina,^2^ optic nerve,^3^^,^^4^^,^^5^ lateral geniculate nucleus (LGN),^6^ or visual cortex.^7^^,^^8^ Retina and optic nerve are potentially interesting implantation choices because of their limited invasiveness and potential performance, while thalamic and cortical approaches are promising for the wide range of diseases they can address.^9^

RS has emerged as leading technology in the vision restoration market,^10^ offering a visual resolution that enables users to perform digit and letter classification.^11^ Several devices have been approved for clinical testing or use,^12^ such as Argus II (Second Sight Medical Products, USA),^13^ Alpha IMS (Retina Implant AG, Germany)^14^ and its successor Alpha AMS,^15^ BVT Bionic Eye System (Bionic Vision Technologies, Australia),^16^ IRIS II, and PRIMA (Pixium Vision, France).^17^^,^^18^

Nonetheless, the portion of the retina that can be covered with a high-density electrode grid is limited by the technology of implantable stimulators and *trans*-scleral connections in traditional prostheses, and by low flexibility in photovoltaic devices, which does not enable conforming to the eye curvature.^19^ The resulting visual perceptions are thus limited to a narrow portion of the visual field: 15° × 15° for Alpha AMS, 11° × 19° for Argus II, 17° × 17° for PRIMA, and 37° × 27° for second-generation suprachoroidal retinal prostheses.^20^^,^^21^

Devices targeting the LGN are currently in pre-clinical development. Their advantages are the possibility of implantation through minimally invasive brain surgery and their potential for application in a broader range of diseases, including glaucoma, macular degeneration, diabetic retinopathy, trauma to the early visual pathway.^12^ However, eliciting discrete phosphenes requires the use of multiple electrodes.^9^

Cortical visual prostheses, as LGN devices, have the potential to address a wide range of diseases, but the implantation surgical procedure is significantly more invasive, and electrode placement is crucial.^12^ Not only must the electrodes be precisely positioned in the foveal region of the cortex to elicit phosphenes in the central receptive fields, but accessing the peripheral visual field is also considerably more complex. This is because the cortical regions corresponding to the peripheral visual field are separated by the interhemispheric fissure that acts as an anatomical barrier.^9^ Cortical devices undergoing clinical trials include ORION (Second Sight Medical Products, USA) and ORION II (Vivani Medical, Inc, USA),^22^ CORTIVIS (Miguel Hernández University of Elche),^23^ ICVP (Illinois University of Technology).^24^

ONS has the potential to produce phosphenes across the whole visual field,^3^^,^^5^ even though their elongated and irregular shapes make it difficult to produce meaningful perceptions and has led to limited application of this approach.^4^ Studying RS and ONS in conjunction may provide particularly useful insight, as they target respectively the somas and axons of the same population of retinal ganglion cells (RGCs). Nonetheless, performing a fair comparison would require the ability to reconstruct the path of each RGC so that the results of RS and ONS refer to the same RGC population. In the present work, we define such a model and characterize its behavior showing its desirable features and limitations, with the aim to determine whether it is possible to produce intelligible phosphene patterns via ONS inside of the constraints of available technology, and which electrode arrangement would be best to this aim.

In the past, ONS stimulation has been mostly administered through extraneural electrodes, which allowed to cover the external surface of the nerve without penetrating it.^4^^,^^5^ Such electrodes are less invasive and lead to less severe foreign body response than penetrating intraneural electrodes, but cannot access deep structures without exciting superficial ones, which would limit in principle the portion of the visual field where phosphenes are elicited. The viability of intraneural electrodes has been shown to produce tactile sensory feedback for prosthetic limbs,^25^^,^^26^^,^^27^ to restore grasping patterns in non-human primates,^28^ and to modulate the activity of visceral organs.^29^ Since the visual field is intrinsically bidimensional, we expect that a bidimensional electrode array will be needed to obtain a satisfactorily coverage. While for the time being only extraneural cuff electrodes^3^^,^^4^^,^^5^ and monodimensional penetrating electrodes^30^^,^^31^^,^^32^ have been implanted in the optic nerve, bidimensional coverage of the cross section of nerves seems a reasonable requirement in the case of visual stimulation. While such electrodes exist in the form of Utah arrays and have been tested in peripheral nerves,^33^ their viability for optic nerve prostheses should be clarified. Here, we do not adopt a specific electrode arrangement. Rather, we show on one hand that idealized bidimensional arrangements of electrodes implanted in the optic nerve can expand the target population of visual neuroprostheses to candidates not viable for retinal stimulation (RS), and on the other hand, that such optic nerve prostheses could in principle rival commercially available RS devices.

To reach our goal, we introduce a method to estimate the best perceptions that can be produced by an electrode configuration to replicate a set of natural visual scenes and evaluate their quality through simulated prosthetic vision (SPV) experiments. SPV experiments are state-of-the-art tools to estimate the effectiveness of different vision restoration strategies, allowing us to evaluate the stimulation outcome without the need for a physical implant.^34^^,^^35^^,^^36^^,^^37^ Natural images need to be processed before being transformed into patterns of stimulation, as the complexity of artificial visual perceptions is heavily constrained by several physiological and technological factors, like the limited number of implantable electrodes, their limited selectivity, and the irregular shape of the produced phosphenes. Such processing is performed by an image processing unit normally integrated in the neuroprosthetic device to extract relevant information from the image stream captured by a camera.^8^ Models of visual electrical stimulation can provide insights regarding the constraints imposed on the complexity of visual perceptions that can be reconstructed,^38^ and help us to infer what information to extract from the original images to optimize the functionality of the restored vision. While early studies focused on structural features like edge extraction^39^ or saliency mapping,^40^^,^^41^ recently deep learning-based processing has gained importance in the field of visual prostheses, thanks to improvement in detection and segmentation techniques.^41^^,^^42^^,^^43^^,^^44^ Specifically, semantic segmentation allows us to identify individual salient objects inside a complex scene and to customize the stimulation based on the context surrounding the user, and their needs.^45^ Here, we used a set of RGB images with an additional channel for encoding depth information (RGB-D images captured in different settings (seated at a table, indoor, and outdoor). We perform semantic segmentation to isolate a number of everyday life objects from the background and apply a grayscale colormap to their masks, based on their depth. This might potentially enable the subjects to perform object recognition based on the shape of the instance, as well as estimate its actual size by exploiting the combined information from its size and color. The stimuli proposed to the subjects are obtained by cascading image processing and the model of electrical stimulation.

Our model allows us to evaluate ONS feasibility while considering the limitation of current technology, and to investigate its advantages with respect to RS in both static and dynamic setups.

## Results

Our work can be divided into two main sections, which will be examined in turn summarizing the employed computational tools, the performed experiments, and their results. In the first part, we introduced a geometrical model of the early visual pathway, and characterized it. In the second, we used such model in conjunction with a simplified method for the estimation of the best visual percepts that can be elicited through a given electrode configuration, and performed SPV experiments to assess the quality of such percepts when the target visual stimuli were obtained through machine learning-based simplifications of natural scenes. Additionally, before performing the SPV experiments, we checked that the visual percepts obtained through our simplified method were compatible with actual electrical stimulation, employing a biophysically accurate model of the optic nerve fibers.

### Characterization of our geometrical model

**Figure 1:**
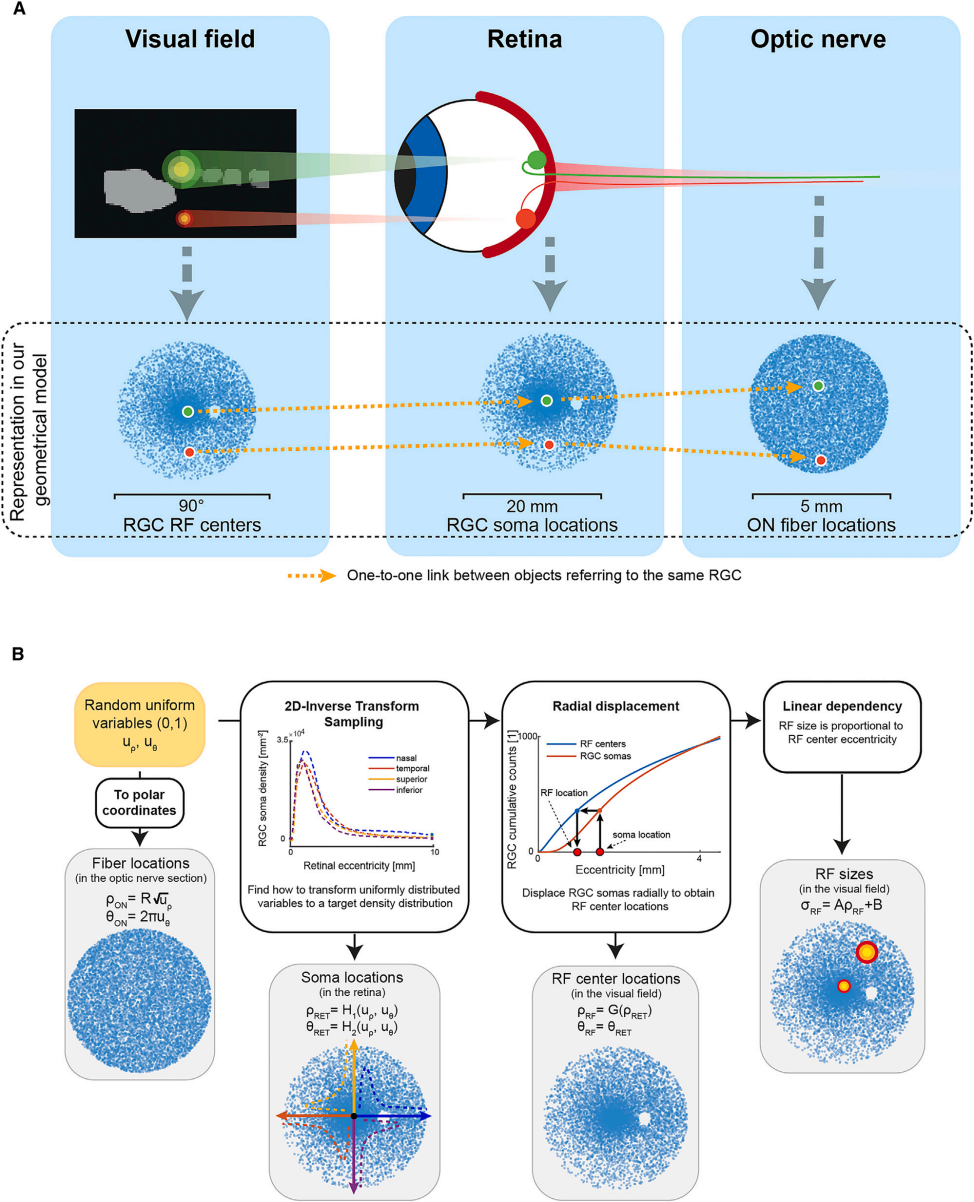
*Graphical representation of our model and method* (A) *Components of our geometrical model*. Scatterplots of the locations of the RGC RF centers in the visual field, of the RGC soma locations in the retina, and of the ON fiber locations in the optic nerve transverse section. Here, we represented pictorially the connection between two RGC RF centers, their soma locations and the locations of the corresponding ON fibers. The chosen RGC RF locations are associated to a simplified representation of their Gaussian isotropic receptive fields with larger receptive field proceeding out from the center. (B) *Generation of RGC locations and RF features*. Sequence of transformations that allow to compute the location of RGC somas, axons and RF features from two-dimensional random variables. Additionally, we show the density distributions for soma locations along the nasal, temporal, superior, and inferior meridians and a schematization of the 2D inverse transform sampling steps; and the method for the displacement of receptive fields from the soma locations and the cumulative distribution curves for the radial distribution of RF centers and soma locations.

In Figure 1A we show how, in our geometrical model, each RGC is represented in three different geometrical spaces: in the visual field, through its receptive field center location and radius; in the retina, through the location of its soma; and in the optic nerve cross-section, through the location of its axon – also referred to as an optic nerve fiber. In the following, we will always assume that receptive field centers, soma and fiber locations are in the visual field, retina, and optic nerve transverse section, respectively.

In Figure 1B, the set of transformations that lead from two dimensionless uniformly distributed random variables in (0, 1) which identify each RGC to its receptive field center location and radius, its soma and optic fiber locations are shown. Since we suppose RGC axons to be uniformly distributed in the transverse section of the optic nerve, the transformation producing their locations from the dimensionless coordinates is straightforward and can be expressed analytically. To determine the locations of the somas in the retina we need to apply 2D inverse transform sampling to the dimensionless coordinates, employing the density measurements provided in Curcio and Allen.^46^ The locations of the receptive field centers in the visual field are obtained from the soma locations in the retina through the method presented in Watson.^47^ All the random variable transformations are detailed in STAR Methods.

#### Our geometrical model includes known anatomical landmarks and a strong retinotopy

**Figure 2:**
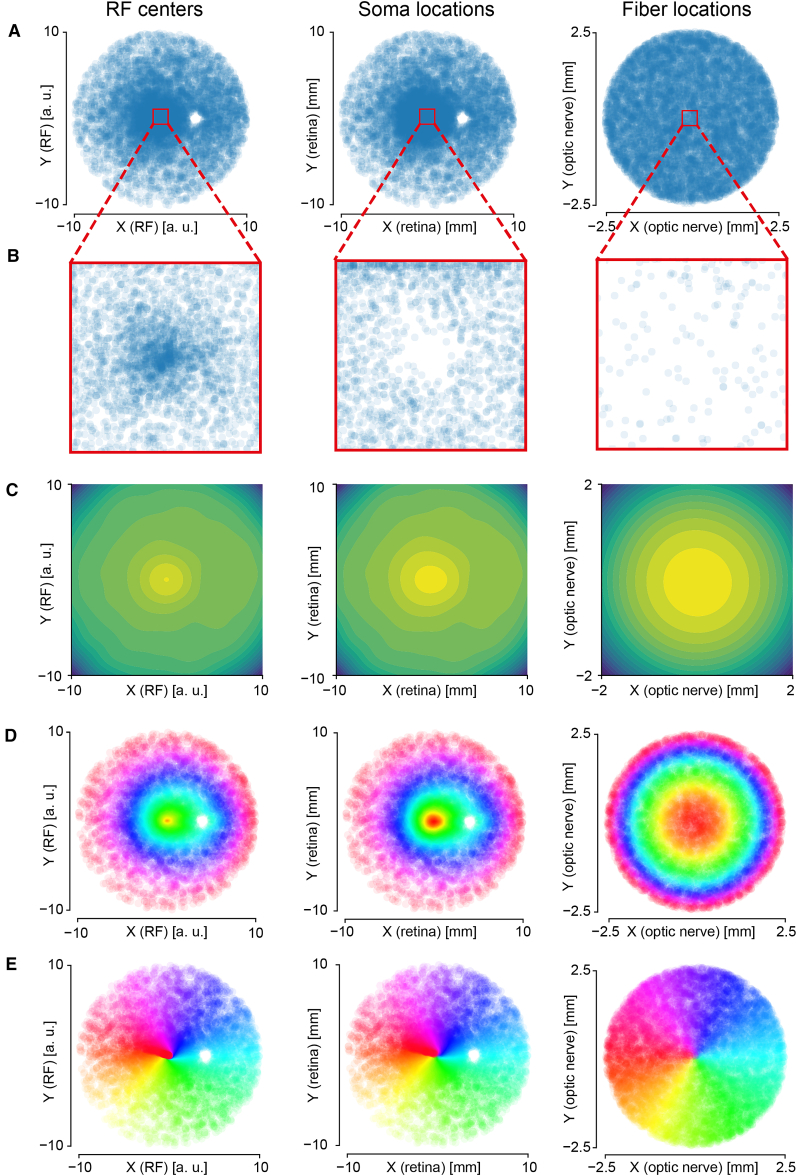
Characterization of the geometrical model (A) Scatterplots of RF center locations in the visual field, soma locations in the retina, and fiber locations in the optic nerve transverse section. (B) Scatterplots of RF center, soma, and fiber locations in a region around the center and size equal to one-tenth of the corresponding geometrical spaces. (C) Contour plot of the density of RF centers, somas, and fiber locations; 10 levels are shown in all three cases. (D) Representation of radial retinotopy. Fibers of different colors correspond to different radial distances from the center of the optic nerve transverse section, the same color is used for each RGC. (E) Representation of angular retinotopy. Fibers of different colors correspond to different angular distances from the positive x-semiaxis of the optic nerve transverse section, the same color is used for each RGC.

In Figure 2A, the distributions of the receptive field center, soma and fiber locations are shown. In the retina there is a circular region depleted of RGC cell bodies, corresponding to the optic disc. Such region is also depleted of photoreceptors, resulting in a blind spot in the visual field.^48^ A zoom in of the macula can be seen in Figure 2B, where it is possible to observe a small region around the fovea that does not contain RGC bodies, while the receptive field density is maximal. This is consistent with the anatomy of the retina, where the fovea is the region with the maximum photoreceptor density and corresponds to the region of the visual field where the highest visual acuity is attained. To accommodate for this very high number of photoreceptors, RGCs are displaced eccentrically producing the very different density profile of their somas in the fovea,^48^ which can be further appreciated by the empirical density plots shown in Figure 2C.

In Figure 2D, the retinotopy properties of the fiber locations assignment between receptive field center, soma and axon locations are displayed. The sequence of colors progresses smoothly in all the plots, indicating that our assignment preserves distance orderings (close-by objects stay closer than far away objects when moving from one space to another). This retinotopy is present both in the radial and angular components of the RGC element locations.

#### The effectiveness of optic nerve and retinal stimulation is limited by geometrical factors

**Figure 3:**
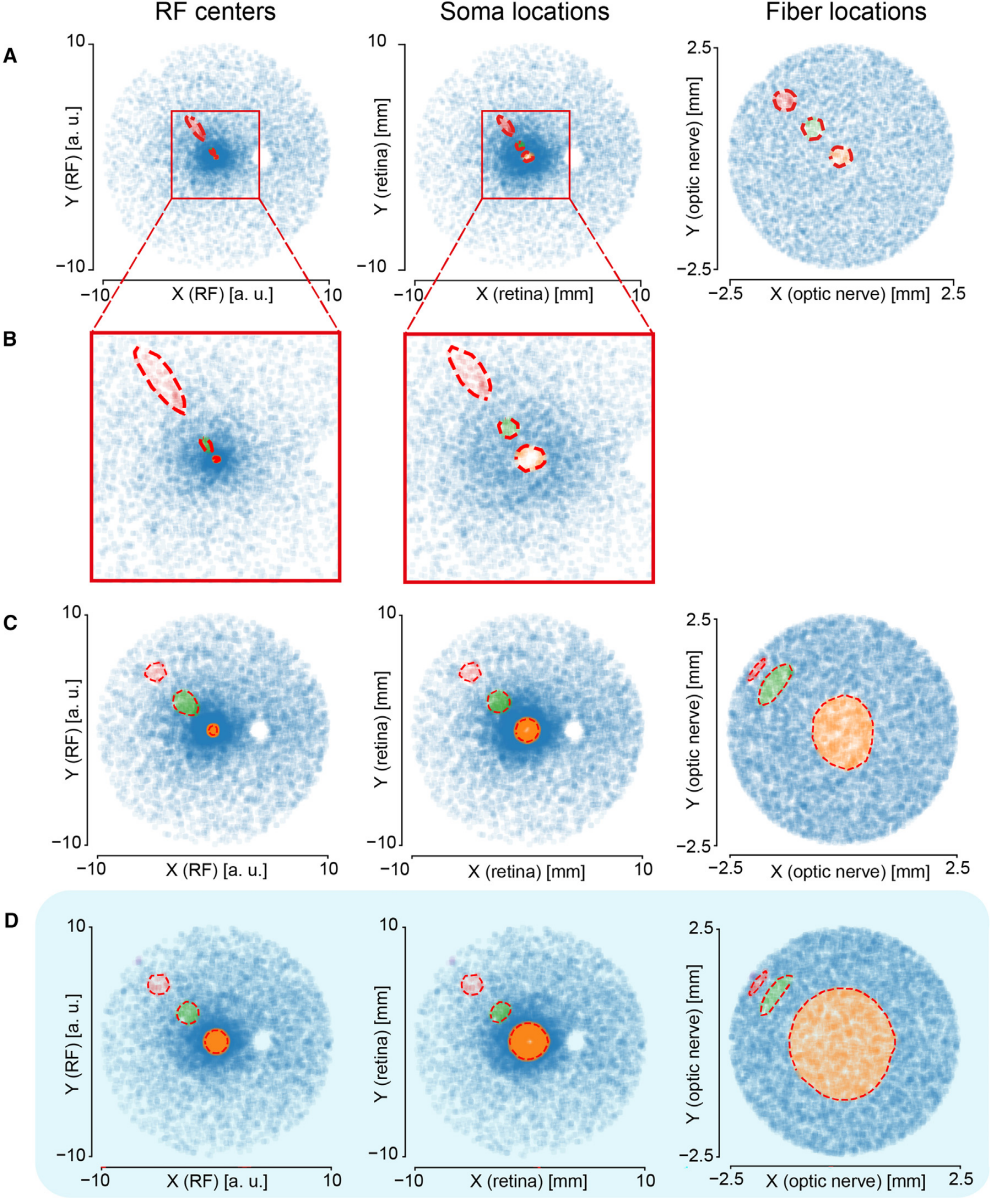
Magnification of a round spot across the different geometrical spaces (A) RFs, somas and fibers associated to circular regions in the ON section. (B) Magnification of the central regions of the first two plots (RFs and somas) from panel A. (C) RFs, somas and fibers associated to circular regions in the retina surface. (D) RFs, somas and fibers associated to circular regions in the visual field.

In Figure 3, we show how circular patches of RGCs in one of the three spaces are mapped to the other two spaces, showing the deformation and displacement that they undergo. As electrical stimulation applied to a homogeneous tissue activates patches of such tissue that are approximately circular, the present figure can give us an estimate of how a stimulation applied to different portions of the retina or the optic nerve corresponds to different phosphenes in the visual field. When electrical stimulation is applied to the optic nerve (see Figures 3A and 3B), the resulting phosphenes become smaller and more circular as the center of the nerve section is approached. Proceeding toward the external surface of the nerve, the phosphenes reach increasingly high levels of eccentricity. Retinal electrical stimulation is also subject to these effects (see Figure 3C), but to a much lower extent and only inside the limits of the so-called displacement zone,^47^ corresponding to a radial distance of 4 mm from the fovea. Finally, it is shown how a circular patch in the visual field is mapped to the retina and the optic nerve (see Figure 3D), which corresponds to the groups of fibers of somas that need to be activated to produce circular phosphenes. While the previous observations still obviously apply, we remark how the RGCs that belong to the fovea occupy a vast area of the optic nerve section, while the peripheral visual field is confined to a very thin ring at the external boundary of the optic nerve.

### Use of the geometrical model to assess and compare ONS and retinal stimulation

**Figure 4:**
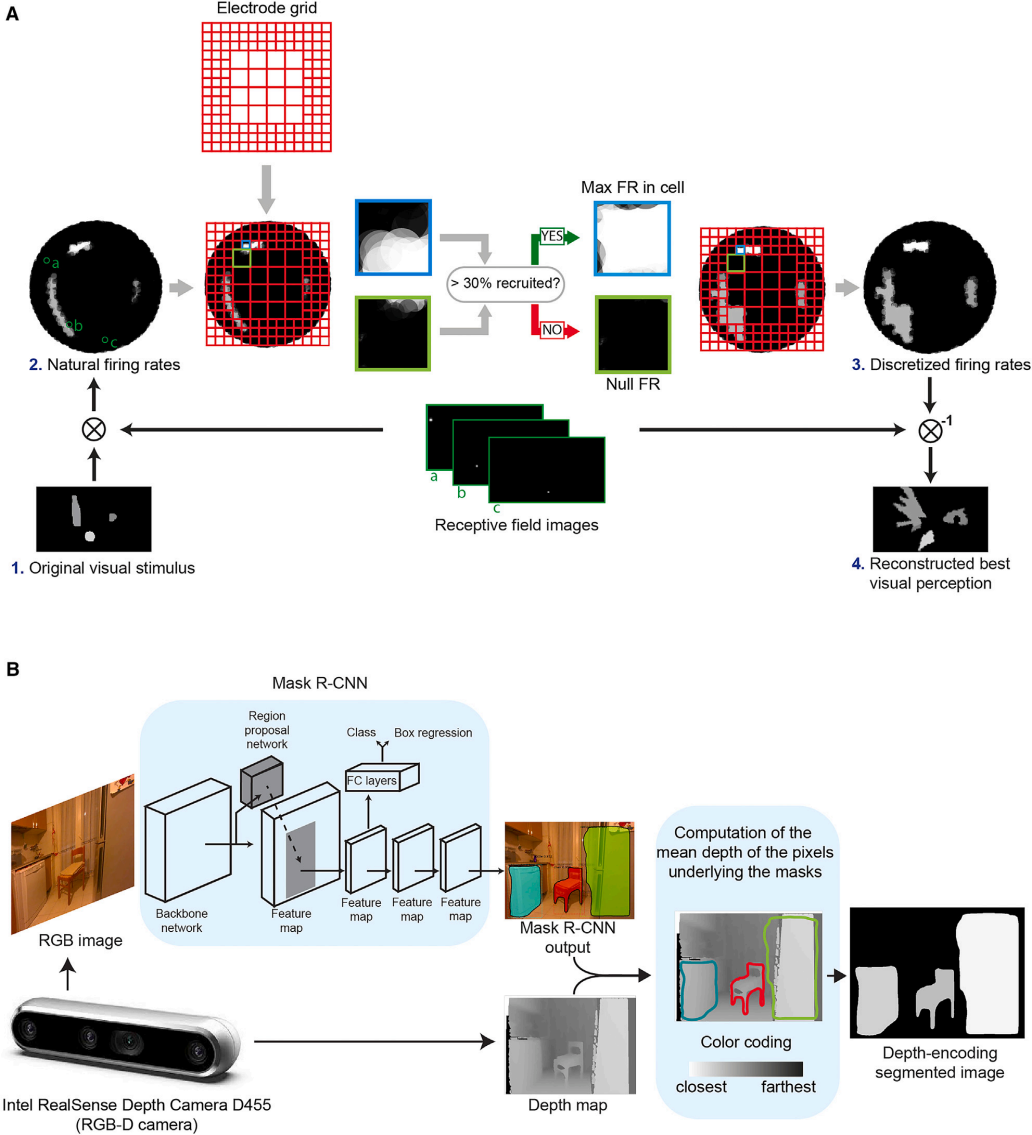
Reconstruction of the best visual perception for a given target visual scene (A) Reconstruction of the best visual perception. A simplified version of a visual scene is converted into a pattern of natural firing rates in the optic nerve through convolution with the RGC RFs. The best artificial firing rate pattern in the optic nerve is determined using a geometrical partition method. Here a red grid represents an electrode, and the fibers within each square are “controlled” by a stimulating site, imagined in the middle of each grid cell. In each cell, the artificially evoked firing rate is set to the maximum firing rate present in the cell if at least 30% of the fibers in its area are active, and to zero otherwise. The resulting pattern is converted back into the reconstructed best visual perception through a deconvolution process using RFs as filters. In the red grids, each square is “controlled” by a stimulating site. (B) Deep learning-based simplification of visual scenes. The RGB part of a visual scene acquired with an RGB-D camera is passed through a Mask R-CNN to perform semantic segmentation. The different object masks are then colored using the depth image acquired from the camera. The depth maps obtained from the camera are then used to color the different object masks, where brightest shades of gray represent the closest instances.

Our geometrical model can be used in the estimation of the best perceptions elicitable, whose workflow is shown in Figure 4A. The receptive field features for one RGC (location and radius) are used to build a “receptive field image”, which determines the sensitivity of the firing rate of the RGC to the intensity of each pixel of a visual scene. From an input image and the receptive field images for all RGCs, we can determine the firing rates of each RGCs. We then introduced a method to estimate from natural firing rates and an electrode array arrangement artificial firing rates that is both compatible with electrical stimulation and is the most similar to the natural firing rate. This artificial firing rate pattern can be converted back into the visual field and be interpreted as the perception that would be elicited on a subject. This method allows us to estimate the best elicitable perception without knowing which stimulation protocol caused it.

Our model was tested on images that have been captured in one of three possible settings: scenes that can be observed while sitting at a table, scenes that can be observed standing indoor, scenes that can be observed standing outdoor. The set of 90 selected scenes is available at [*a link will be made available upon acceptance*].

**Figure 5:**
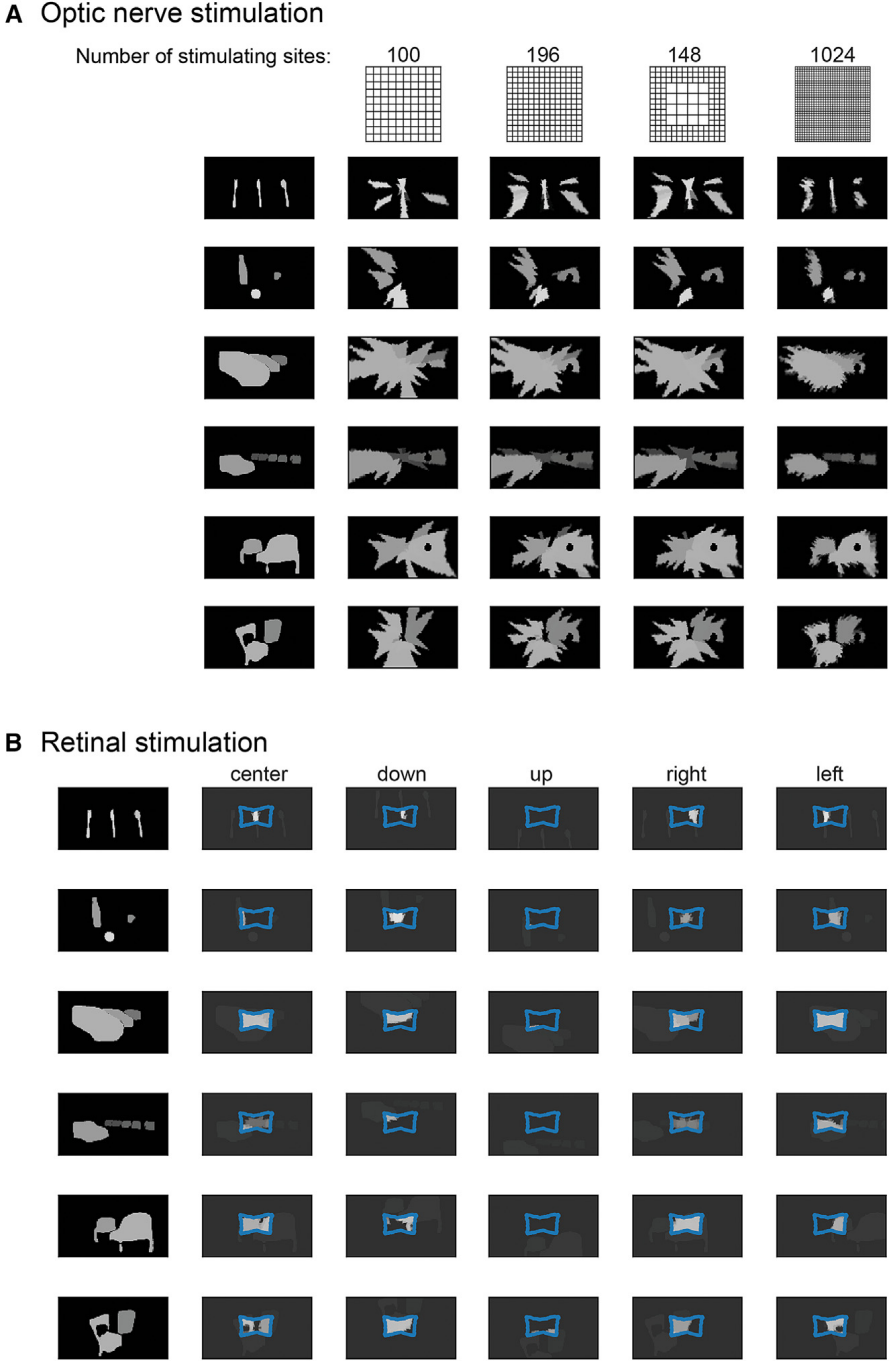
Optic nerve and retinal simulated prosthetic vision (A) Best reconstruction of different visual scenes, obtained using different electrode configurations, with an electrode count between 100 and 1024 electrodes. (B) Reconstructed scenes obtained through simulated prosthetic vision; the blue outline includes the portion of the retina covered by the electrode array. The reconstructed scenes are overlaid on a transparent copy of the undistorted stimulus mask. The original images are either left in the original position of the visual field, or have been displaced 5 pixels down, up, right or left. The first columns in both panels contain the undistorted stimulus masks.

Since natural visual scenes exhibit very complex patterns of luminance and contrast, applying our partition approach to them without further preprocessing results in very confused perceptions (Figure S1). We thus decided to reduce the amount of information that is carried by each natural scene by selecting only a set of salient objects through semantic segmentation and by assigning to the region of the visual field that they occupy a gray level, which corresponds to their average distance from the subject following a conversion law specific of the given setting. The whole method is visualized in Figure 4B. The scenes tested in this work were assigned to a category before processing, but it is possible to automate this choice by integrating a convolutional neural network that classifies scenes into “table”, “indoor” and “outdoor” scenes. The classification performance is displayed in Table S1. Further details on the instrumentation and algorithms employed can be found in Methods. The resulting grayscale processed images are available at [*a link will be made available upon acceptance*] (a subset of them can be found in Figure 5).

#### Our geometrical model can be used to evaluate different electrode configurations

In Figure 5A, we show the best reconstruction of different visual scenes obtainable using different electrode configurations. We can see that in general a very high electrode count is required: already with a 10 × 10 electrode grid, the resulting reconstructed scenes are heavily distorted. The shapes and relative sizes of the objects are strongly affected, and small objects cannot be captured. When we increase the number of electrodes, we see that all these problems are mitigated, even though we need unreasonably high electrode counts to obtain visually appealing results (see the reconstructed images obtained with 32 × 32 electrodes). By exploiting the previous observations on the magnification of the foveal region from the visual field to the optic nerve section, we can reduce the number of electrodes placed in the center of the optic nerve, and still produce acceptable visual scene reconstructions. In Figure 5A, we show how we can reduce the number of stimulating electrodes from 196 to 148 (a reduction of almost 25%) substituting 2 × 2 electrode sub-grids in the center of the optic nerve/visual field with single electrodes placed in the center of the grid, without substantial degradation of the reconstruction of natural visual scenes. The rationale is for this electrode arrangement transformation is to decrease the number of independent degrees of freedom/stimulating electrodes placed in the center of the visual field, where the effect of magnification is larger and the visual field portion covered by single electrodes is smaller. In Figure 5B, the reconstructed scenes using the 60 (6 × 10) electrode Argus II epiretinal prosthesis are shown, for different locations of the center of the visual field in the scene. While RS allows us to attain good resolution in the center of the visual field (Figure S2), it seems inappropriate for the reconstruction of static, wide-field scenes. At the same time, the very poor resolution that is provided by ONS, especially in the center of the visual field, makes it meaningless to put any technological effort in relying on increasing angular resolution to the level of providing useful percepts of minute features rather than pushing toward low definition large-FOV perceptions. Finally, to further characterize the behavior of our geometrical model, we show in Figure S3 the reconstructed visual scenes employing a 14 × 14 electrode grid covering the whole surface of the retina, similarly to what has been done for the optic nerve. Employing similar electrode grids for RS and ONS produces similar visual resolution but much higher phosphene elongation/deformation in the case of ONS.

#### Our simulated percepts are compatible with biophysical models of electrical stimulation

**Figure 6:**
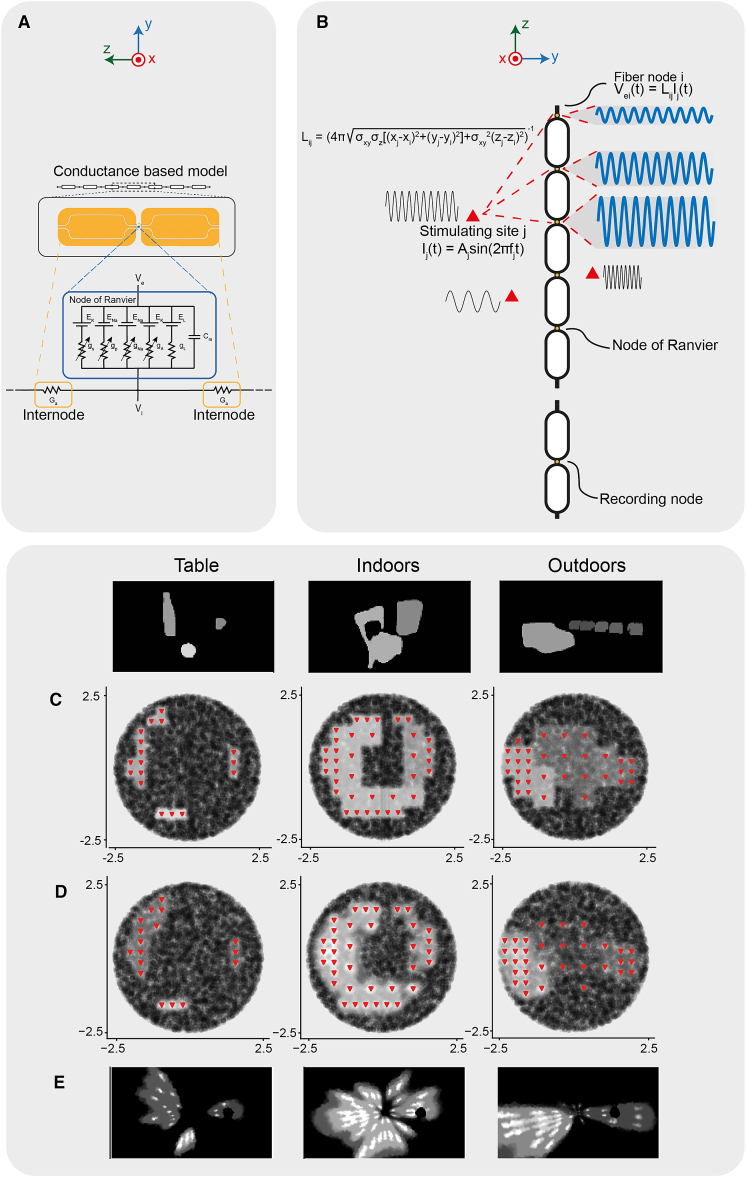
Biophysically accurate model of optic nerve fiber stimulation (A) Equivalent circuit of a Ranvier node and two neighboring internodes for the employed optic nerve fiber model. (B) View of the longitudinal path of an optic nerve fiber and of the stimulation configuration. The extracellular potential at each Ranvier node of the fiber is computed through the linear combination of the current applied by each stimulating site and the corresponding entry of the lead field matrix. (C) Firing rates obtained using the geometrical partition method, used to generate the visual stimuli that were delivered to the subjects in this study. (D) Firing rate obtained in the optic nerve using the biophysical model to simulate a 1,000 ms stimulation on the full population of 10,000 fibers. (E) Reconstruction of the stimuli based on the simulated firing rate of the full 10,000 fibers population. Each column corresponds to a single visual stimulus, shown in the first row. The red triangles are the active sites.

We implemented a biophysical model of optic nerve electrical stimulation to show that our geometrical method produced reasonable simulated percepts. In other words, to demonstrate that the introduced approximations do not lead to percepts substantially better than what would be obtained through actual electrical stimulation. We implemented a model of optic nerve fibers from the literature^49^^,^^50^ using the NEURON simulation environment (Figure 6A), applied a pattern of extracellular potentials to such model and computed the firing rate as the number of spikes over the duration of the stimulation. We computed applied sinusoidal currents with given amplitude and frequency, under the assumptions that the electrode sites are isotropic point current sources placed in the center of our grid cells, and that the nerve is a linear, purely resistive, homogeneous, isotropic and infinite medium (Figure 6B). Further details are presented in Methods. We manually tuned the amplitude and frequency of stimulation for each electrode until we obtained satisfactory percepts, which are displayed in Figure 6E. In Figure S4, the electrode-wise amplitudes and frequencies of stimulation are reported. The simulations have been performed on both a subsampled population of 2,500 optic nerve fibers, simulating a 500 ms stimulation (Figure S5), and on the full 10,000 fibers population, simulating a 1,000 ms stimulation (Figures 6C–6E). The execution times were 190 s (table), 192 s (indoors) and 190 s (outdoors) in the subsampled-population simulation, and 1,366 s (table), 1,457 s (indoors) and 1,381 s (outdoors) in the full-population simulation. Visual inspection of the resulting percepts showed that they are indeed compatible and that our approximations in the geometrical model did not produce physically unfeasible reconstructions.

#### Geometrical models can be combined to simulated prosthetic vision to compare different stimulation paradigms

**Figure 7:**
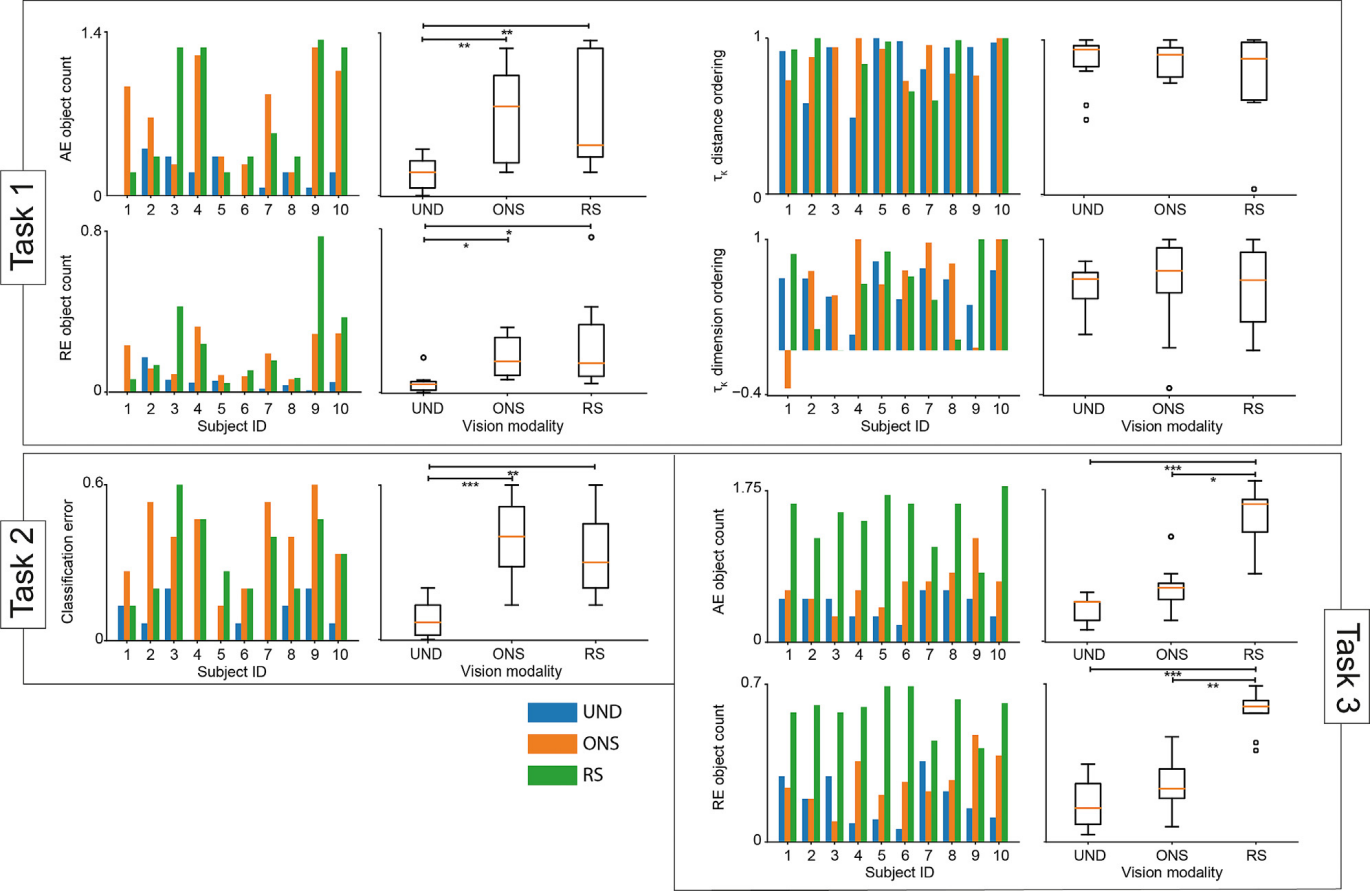
Results of simulated prosthetic vision experiment (Task 1) Absolute and relative errors in object count, and Kendall’s tau for distance and dimension ordering. (Task 2) Object classification errors. (Task 3) Absolute and relative errors in object count. In all cases, we show a bar chart with subject-wise data (subjects 1–10) and boxplots with data aggregated by vision modality (UND: undistorted, ONS: optic nerve stimulation, RS: retinal stimulation). Asterisks indicate statistical significance from post-hoc Dunn test with Bonferroni correction, performed if Kruskal-Wallis test resulted significant (*p* < 0.05); asterisk legend: ∗*p* < 0.05, ∗∗*p* < 0.01, ∗∗∗*p* < 0.001.

We performed a set of SPV experiments on a group of ten healthy subjects to assess whether ONS could be a viable technology and to compare it with a commercial RS technology in a more realistic setting. During the experiments, the subjects observed and were able to navigate static and dynamic scenes reconstructed according to retinal and optic stimulation and answered to several questions assessing the quality of the reconstructions. For RS, we employed reconstructions corresponding to the Argus II interface, while for ONS we employed the inhomogeneous 148 electrode grid represented in Figure 5A.The experiment involved three tasks. In the first task, the subjects were asked to report how many distinct objects were present in a set of static scenes, to rank the objects in order of increasing distance, and to rank the objects in order of decreasing dimension. In the second task, the subjects were asked to focus on one object in each one of a set of static scenes and to classify it choosing from a set of three possible labels. In the third task, the subjects were asked to count the number of objects appearing in a dynamic scene. The dynamic scenes proposed during the experiments are available at [*a link will be made available upon acceptance*]. Further details on experimental design and on the modality of administration of the visual stimuli are provided in Methods. In Figure 7, the results of the experiments are presented in terms of the subject-wise bar charts for every task, and of the boxplots aggregating all subjects to compare their performance on the undistorted images and on the reconstructions obtained using simulated optic nerve and RS, respectively. We notice how subject-wise performance differed heavily. The results of the comparisons between undistorted, retinal and ON reconstructions are reported in Table 1. As expected, the performances on the undistorted images were significantly better than those on the simulated prosthetic vision in all cases. ONS and RS results were not significantly different in tasks performed on static scenes, but ONS performed significantly better when dealing with dynamic scenes. In addition, a total of 10 virtual experiments were conducted in which random answers were simulated, to assess the difference from the actual subjects’ performance. More details on simulation of experiments and generation of random answers are reported in Methods. The first quartile, median, and third quartile of the random performance are reported in Table 2. These results show that, although ONS and RS do not allow subjects to achieve the same performance achieved with undistorted images, they provide subjects with enough visual information to improve their answers compared with chance level.

**Table 1:** Results of the comparisons of the performance between undistorted, retinal and ON reconstruction

–	ON	RET
Static framework		
Absolute error in object count		
UN	0.004	0.004
ON	X	1
Relative error in object count		
UN	0.02	0.01
ON	X	1
Kendall’s tau for distance ordering		
UN	1	1
ON	X	1
Kendall’s tau for dimension ordering		
UN	1	1
ON	X	1
Object classification error		
UN	0.0005	0.005
ON	X	1
Dynamic framework		
Absolute error in object count		
UN	0.31	0.00002
ON	X	0.01
Relative error in object count		
UN	0.86	0.00006
ON	X	0.004

**Table 2:** Performance of the simulated random experiments

–	Q25	Q50	Q75
Static framework			
Absolute error in object count	1.2	1.5	1.58
Relative error in object count	0.64	0.74	0.88
Kendall’s tau for distance ordering	−0.38	0.05	0.33
Kendall’s tau for dimension ordering	−0.33	0.05	0.38
Object classification error	0.22	0.33	0.38
Dynamic framework			
Absolute error in object count	0.9	1.0	1.1
Relative error in object count	0.38	0.47	0.51

#### Factors that may affect the quality of elicited visual perceptions

**Figure 8:**
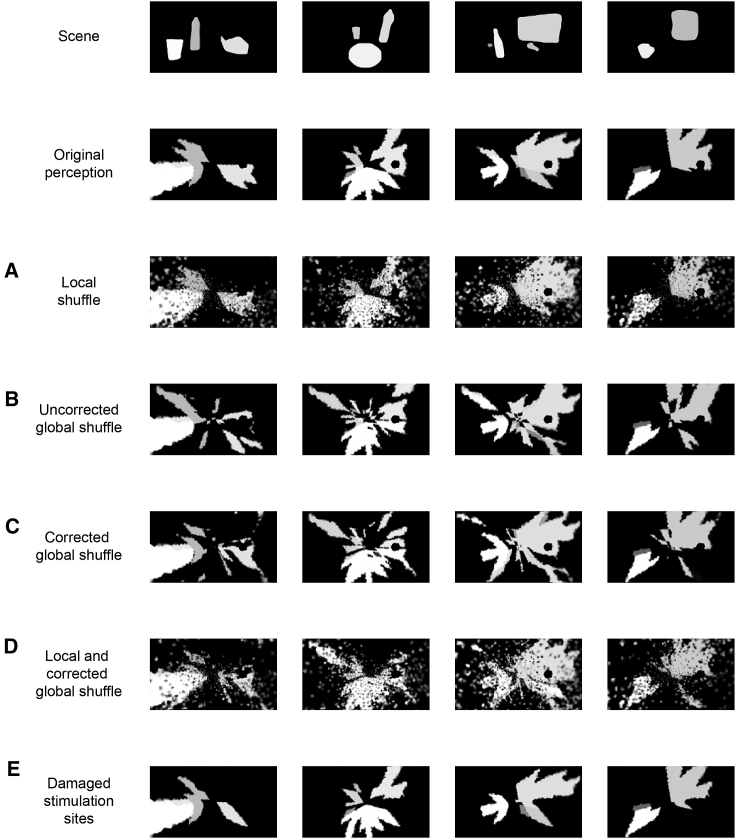
Factors that may affect the quality of the reconstructed perception (A) Effect on the chosen scenes of local shuffling of RGCs. (B) Effect of uncorrected global shuffling of RGCs. (C) effect of global shuffling corrected through the identification of the electrode contacts targeting each RGC group. (D) joint effect of local shuffling and corrected global shuffling. (E) effect of malfunctioning (broken) stimulation electrode sites.

In this section, we describe the effect of three factors affecting the quality of the visual perceptions obtainable through our framework, degrading it from the ideal case: imperfect retinotopy, implanted electrode malfunctioning, and RGC axonal degeneration. Here, we model imperfect retinotopy through the superposition of two distinct “disordering” mechanisms. Macroscopic portions of the optic nerve fiber population can be displaced from the location the corresponding somas occupied in the retina, similarly to what is observed at the level of the optic disc where the macula is located laterally, producing a “global shuffling” of the fibers. Because it is not reasonable to assume that fibers follow these global rearrangements through “parallel flow”, a local shuffling/disordering has been added on top of the above mentioned global shuffling. The introduced local shuffling (displayed in Figure S6) has the effect of producing heavy salt-and-pepper noise as can be seen in Figure 8A, caused by the mismatch between the high spatial correlation of the firing rate patterns elicited in close-by fibers by electrical stimulation and the introduced disordering. While the imperfect selectivity of current interfaces limits the ability to cope with local fiber shuffling, global shuffling can be partially corrected, as can be seen comparing Figures 8B and 8C. The process of experimental mapping of the regions activated during stimulation is outlined in Figure S7. Each electrode is activated in turn, and the phosphene pattern described by the patient are referred back to the retinotopic map of the nerve, thus inferring the global shuffle that generates the patient-specific retinotopic mapping. Repeating for each available stimulating site, we manage to establish the map from the retinotopic geometrical model to the personalized non-retinotopic one. Such correction does not compensate global shuffling completely though, because of the mismatch between the subdivision into zone of influence of the different electrodes and the subdivision into different fiber subpopulations. In Figure 8D, we show the degradation produced when both local shuffling and corrected global shuffling are concurrently present. The effect of having a set of malfunctioning electrodes is presented in Figure 8E: while a large portion of electrodes was inactivated, the recovered perceptions retained acceptable quality, since the processed images are heavily simplified. Finally, in Figure S8 we provide a prediction of what would happen in cases of severe degeneration of optic nerve fibers. In such cases, the visualization of the predicted outcome of stimulation is particularly useful to decide whether ONS may be a viable alternative for the target patient, or whether the large damage at the level of the optic nerve would prevent most of the benefits of this approach, choosing instead for the patient a cortical prosthesis.

## Discussion

In the present work, we introduced a geometrical model that associates the locations of the receptive fields, somas and axons of RGCs at the levels of the visual field, retina and optic nerve (Figure 1). This model allows the conversion of visual perceptions/stimuli into spatial arrangements of firing rates at the levels of the retina and optic nerve. We coupled our model with a method to estimate an upper boundary on the quality of the percepts generated by a given stimulating electrode configuration (Figure 4). Thus, our method can be used to compare electrode configurations for the stimulation of the optic nerve, and to determine the most suitable one.

Here, we considered a very strict definition of retinotopy, namely the fact that RGCs that have higher values of the radial or angular coordinates in the retinal space, have also higher values of the radial and angular coordinates in the optic nerve section (Figure 2). Such strict retinotopy derives by the fact that both the transformations applied via 2D inverse transform sampling and by the displacement between RGC somas and receptive fields are smooth. In the limitations section, we will discuss how reasonable it is to assume retinotopy and how we can control the extent of imposed retinotopy.

To build our geometrical model, we employed human cadaveric data. Nonetheless, the presented method to generate smooth retinotopic maps between arbitrary distributions can be employed with any kind of experimental data. For example, in Figures S9 and S10, we show that it is possible to implement the map between homogeneous distribution of optic nerve fibers and animal distribution of retinal ganglion cells in the retina from.^51^^,^^52^ Our method can be also employed to map a given distribution of RGCs in the retina to an inhomogeneous distribution of nerve fibers in the optic nerve. In Figures S1 and S12, we provide simulations including the slight density gradient present in the human optic nerve,^53^ showing that does not alter substantially the resulting visual perceptions and may thus be neglected in future analyses.

Our model suggests the presence of purely geometric factors that define the main challenges to be addressed to develop effective ONS, justify phenomena observed in experiments, and guide the development of novel ways to stimulate optic nerve and retina (Figure 3). For example, it provide intuitive justification for the very elongated phosphenes produced by cuff stimulation of the optic nerve,^3^ as can be seen from Figure 3A.

The best reconstruction of the scenes obtained simulating different grids of electrodes show that a grid of 14 × 14 stimulating sites, is already sufficient to produce understandable images. Increasing the number of implanted stimulating sites also increases the invasiveness of the electrode array, and the proportion of tissue undergoing foreign body response and degeneration. While this matter requires particular attention, and it should be the object of future in silico and experimental studies, in Figures S13 and S14 we show the effect of the formation an encapsulation tissue with radius 50 or 100 μm (typical values found in literature for implanted electrodes^54^^,^^55^^,^^56^) depleted from nerve fibers around each electrode site. The very high proportion of cell death is coherent with the fact that the proposed implants are in general more invasive than the standard Utah array, with inter-electrode distances of approximately 360 μm (10 × 10), 260 μm (14 × 14), and 110 μm (32 × 32), while the standard Utah array has an inter-electrode distance of 400 μm.^33^^,^^57^ Additional SPV experiments (Figure S15) confirmed that the relative advantage in object recognition abilities granted increasing the grid arrangement from (14 × 14) to (32 × 32) is smaller than the gain from a (10 × 10) to (14 × 14) arrangement (8% gain in object recognition accuracy from a 96% increase in electrode count in the latter case, while only 18% gain from an increase >400% in electrode count in the former case). This confirms that although finer grids and thus higher visual resolution increase object recognition abilities, this benefit does not justify a strong push on the technological side.

The geometrical analysis of the magnification between different spaces can be exploited to reduce the number of stimulating electrodes in an electrode array. For example, since the stimulation of the central optic nerve fibers results in very small and circular phosphenes it is possible to reduce the number active sites in the central optic nerve section without causing substantial degradation of wide-field visual scenes. In our model, the difference between images reconstructed using 196 and 148 active sites is minimal (Figure 5A). Such an arrangement of active sites could be reached by exploiting several rings of radially distributed penetrating needles, organized in several shafts placed longitudinally along the nerve, similarly to what is proposed in.^58^ Irregular arrangements of stimulating sites could be developed for RS as well, matching the distribution of RGC somas or taking into accounts the shape of the produced phosphenes.^59^ Still, the main bottleneck to increasing spatial resolution is given by crosstalk, which is not addressed by these attempts. While the number of stimulating sites could be likely decreased, to leverage this advantage to increase the targeted surface of the retina the main issue of the conformability of the implanted array to eye curvature would remain. While RS results in general into a much higher resolution than the one that we can hope for in ONS, the narrowing of the field of view makes it fundamentally impossible to reconstruct complex scenes without performing a thorough visual exploration (Figure 5B), which greatly limited the usefulness of simulated RS in highly dynamic environments.

Then, we checked that the quality of the perceptions obtained through our geometric partition method was not substantially better than what can be obtained via validated optic nerve fiber biophysical models (Figure 6). In particular, crosstalk between stimulating sites could have hindered the ability to control independently the firing rates of the fibers in different electrode cells. We have shown that this is not the case. The only qualitative difference between geometrical and biophysical perceptions are the small white spots that can be noticed around the active sites in the biophysical ones. Such bright spots, however, are very close to stimulating sites, in regions that *in vivo* should be devoid of nerve fibers because of the foreign body response close to the implanted stimulating sites, which should decrease the strength of such bright spots.^56^

Interestingly, our psychophysical experiments showed a huge variability between subjects (Figure 7; Tables S2–S4). When performing tasks on static scenes, some subjects attained better results with simulated RS, and others with ONS. Aggregating the results from all subjects, we obtained that RS and ONS do not produce substantially different performances. Distance ordering was easier to perform than dimension ordering. This is reasonable, as distance is immediately encoded into object color, while absolute object dimensions had to be guessed exploiting their size in the image with their distance coded by color. Interestingly, the performances in distance and dimension ordering were comparable for distorted and undistorted vision modalities, indicating that the key information to better estimate dimension is already lost stepping from natural images to masks. Instead, in dynamic scenes, the performances in the case of retinal prosthetic vision stimulation were significantly worse with respect to both undistorted images and optic nerve prosthesis simulations (Table 1). This is most likely due to the narrow field of view provided by retinal prostheses^20^ In fact, it has been shown that the minimum functional field of view diameter in humans is around 30°,^60^ and Argus II^13^ retinal prosthesis, which was simulated in this study, does not satisfy this requirement, with a 11° × 19° FOV.^20^ The FOV size has a great influence on the quality of vision, although research has not focused extensively on this factor.^60^ Although in this study it particularly affected subjects’ performance in dynamic contexts, it was shown that in some cases adequate FOV size reduces the influence of resolution on prosthetic vision.^61^ The obtained results are substantially better than random guessing in all conditions except for object counting in dynamic scenes under simulated RS (Table 2). This suggests that while both RS and ONS produce some kind of functional vision restoration in static settings, RS may not be enough to produce functional vision restoration in highly dynamic settings. It is important to notice that wide-FOV photovoltaic retinal prosthesis are being actively developed, see for example the POLYRETINA,^62^^,^^63^ but they are still far from being fully characterized in patients and they are limited by their intrinsic inability to provide arbitrary stimulation protocols. Finally, the development of more refined SPV designs based on virtual reality,^61^^,^^64^ where subject proprioception could help them orient in space and potentially reduce the impact of limited FOV.

The proposed geometrical model constitutes an ideal starting point for the development of more accurate and personalized models that can help optimize evoked perceptions in specific target patients. In Figure 8, we provided a limited set of examples of the possible causes of distortion that can act on top of the ideal retinotopic model to produce subject-specific sensations. The definition of more accurate personalized models needs to go at the same pace with experimental advances allowing the measurement of the new parameters introduced to modify the ideal model. Concurrently, cadaveric studies can provide the basis for parametric analyses of robustness, leading to the development of stimulation optimization routines that are less sensitive to inter-subject variability.

The feasibility of ONS has been demonstrated in several studies, but its use has not been widespread, mostly because of the elongated and irregular shape of the produced phosphenes, which make it difficult to elicit useful visual perceptions. Here, we presented a geometrical model that allows us to associate firing patterns in the retina and the optic nerve and to reconstruct the visual perceptions generated by such firing patterns, enabling the characterization and comparison between RS and ONS. Our geometrical model provides reconstructed visual perceptions which are similar to those that can be obtained through detailed biophysical models of electrical stimulation. We exploited our model to show that one of the main advantages of stimulating the ON using a grid electrode would be the possibility for the subject to be aware of dynamical objects whose movement spans the whole visual field. Our model will be used in conjunction with advances in hybrid models of neuromodulation to pave the way for automatic optimization of ONS.

The present work sets the basis for a direct comparison of ONS and RS, particularly interesting for the patients who could in principle benefit from both approaches. While in the present work we did not model the phosphene elongation produced by the activation of passing RGC axons, which would worsen the quality of our simulated RS-produced percepts, it seems evident that ONS will produce percepts with a much lower spatial resolution when administered through existing technologies. This may hinder tasks like reading but could be enough for navigation tasks, even at lower electrode counts, which are in the reach of current technology. The main advantage of ONS is the ability to cover the whole visual field with a number of electrodes and technology that is currently available, while RS will need substantial technological leaps to achieve comparable performance on this respect. This would increase the awareness of the surrounding environment in implanted subjects. More immersive and dynamic SPV experiments employing our model and methods should be developed to further evaluate the functional gain stemming from that.

### Limitations of the study

In the presented model, the locations of the RGCs axons in the optic nerve show excellent retinotopic organization, both in the radial and angular directions. This choice is supported by some indirect evidence: past ONS experiments seem to suggest retinotopic organization close to the optic chiasm,^3^^,^^5^ and studies on mice reported that there is a partial recovery of dorsoventral retinotopy at the chiasm level.^65^ Indeed, it is reasonable that the portion of the optic nerve closer to the optic chiasm may exhibit a retinotopic organization, since both the LGN^66^ and the sensory cortices V1 to V4^67^ display strong retinotopy. Nonetheless, one of the main reasons to try to reach perfect retinotopy in a model like the one introduced in the present work is that once perfect retinotopy is available, algorithms to map to experimentally characterized retinotopic organizations can be easily implemented, while the converse does not seem as straightforward.

Unfortunately, to the best of our knowledge, no detailed direct accounts of optic nerve retinotopy can be found in the literature. Qualitatively, it is known that the macula is projected to the most temporal portion of the optic nerve head,^68^ resulting from the paths that RGC axons follow from their soma to their entry in the ON at the optic disc, which have been modeled in Jansonius et al.^69^ These accounts also suggest that while there may be a macroscopic reorganization of RGC locations, close by RGCs should stay close in moving from the retina to the ON, thus maintaining a strong “local retinotopy”. This local retinotopy may disappear proceeding away from the eyeball, as shown in cats in Horton et al.^70^ There, optic nerve fibers diverged quickly from a retinotopic arrangement behind the eyeball, and maintained their position in the nerve section along the rest of their path. The amount and type of fiber reorganization can influence differently the stimulation outcome: for example, long-range displacements of large “homogeneous” patches of fibers will not pose a challenge, while short-range mixing of fibers inside such patches will inevitably limit the quality of the corresponding sensations because of the imperfect selectivity of current neural interfaces.

We further hypothesized the relationship between RGC firing rates and brightness in their receptive field to be linear, which ignores the static nonlinearity and time-dependencies typically determined from experimental recordings from RGCs.^71^ In future models, these non-linearities/non-stationarities should be included to increase the biophysical detail of the present simulations. In any case, time-dependencies in the response to electrical stimulation can originate either at the level of stimulation (because the targeted cells exhibit habituation to a stationary pattern of stimulation), or through adaptation in the downstream processing. Since both RS and ONS act on the firing rate produced at the level of RGCs, all downstream processing steps are shared, and we expect that introducing time-dependencies will affect equally RS and ONS. As for adaptation at the level of the targeted population, both in epiretinal RS and ONS the targeted structures are the axons of the RGCs, and thus time-dependencies will be similar in the two cases. It is worth noticing, though, that when moving to subretinal and suprachoroidal the increased engagement of retinal circuits will likely introduce additional time-dependencies. This will potentially further degrade the quality of our estimates for RS, for which we have now provided optimistic estimates. Future studies will need to clarify these aspects.

Here, we have manually set the stimulation parameters to obtain visual perceptions similar to the target scene. Biophysically detailed simulations have been performed using the hybrid modeling framework^72^ with the usual quasi-static approximation^73^ for the computation of the stimulation lead field matrix, but including the time dynamics of the fiber response to electrical stimulation.^49^^,^^50^ As we said in the above paragraph, we ignored in this first work on the topic the temporal dynamics of the conversion between firing rates and perceived brightness. The process of stimulation protocol optimization should be automatized using techniques like genetic algorithms or particle swarm optimization, like shown in Romeni et al.^74^^,^^75^ To reduce the computational cost of evaluating the effect of the applied stimulation on the target nerve fibers, surrogate models capturing the behavior of biophysical models without including all their computational details should be used. While fiber activation can be predicted at a low computational cost using model-based surrogate models like the activating function formalism,^76^^,^^77^ these methods are based on linear cable theory and do not generalize to the prediction of firing rates, which depend upon inherently nonlinear phenomena. Machine learning-based surrogate models like the ones shown in^75^^,^^78^^,^^79^ should be explored to this aim.

In the present work, we compared an idealized scenario of ONS to a commercial device to perform RS. This choice should not be read as an attempt to provide a positively biased evaluation of the capabilities of ONS with respect to RS. Rather, it should motivate the investigation of ONS in parallel with new RS approaches. ONS has never been tested on multiple patients and holds the potential to rival in specific settings with commercially available devices for RS, which should motivate its exploration. The research on ONS would also target a portion of patients who could not benefit from RS because for example affected by retinal degenerations that are too severe. In the future, geometrical and even biophysical models of several approaches to RS should be developed to carry on a fairer comparison. Here, we have evaluated ONS applied through a small set of abstract electrode array configurations. It would be interesting for future studies to determine the optimal electrode layout through a proper optimization process that could consider both the performance of the electrode configurations and the invasiveness of the proposed setup.

The simple SPV experiments that we presented may be improved in the future by constraining the head-to-screen distance and performing eye tracking of the subjects, to produce more controlled visual stimulation scenarios. As a further step, immersive virtual reality could be employed, where eye tracking is employed not only to check *a posteriori* the direction of gaze of the subjects, but it can allow the online computation of the scene projection to be proposed to the subject. In addition, immersive virtual reality may allow the simulation of more realistic and interactive functional tasks. Here, we privileged a simple setup with the main goal of providing some first evidence that ONS can be a promising stimulation technique, and to encourage future experimental analyses to quantify more precisely its actual potential. Building more constrained setups or virtual reality ones requires solving a number of technical challenges related to the subjects’ comfort and the possibility of cyber-sickness that go well beyond the scope of the present work.

An interesting variation of the proposed SPV experiments could include subject-specific geometrical models. Here, the fact that different subjects performed better in static recognition tasks with scene distortions corresponding to RS or ONS were attributable to subjective differences in the interpretation of the same sets of visual stimuli, since the same geometrical model parameters were employed for all subjects. Through cadaveric studies, the inter-subject variability of physiological quantities like the distributions of photoreceptors, RGC somas and optic fibers, as well as the extent of retinotopy could be measured. Proposing to the healthy subjects’ scenes reconstructed using different but plausible geometrical models would allow to study the robustness of the proposed vision restoration approaches to the inter-subject variability that is expected in patient populations.

In the future, a biophysically accurate model of RS should be defined and implemented. In the case of the retina, the response of the RGC is modulated both by the applied electrical stimulation and by the surviving retinal cell network. Moreover, while optic nerve fibers can be modeled as unbranched multi-compartmental cable models, the modeling of retinal cells requires dealing with morphologically complex neural models. One possible framework for the development of a biophysical model of RS is the so-called hybrid modeling,^72^ cascading the computation of the electric potential field across the retinal tissue under electrical stimulation (the volume conduction problem), with the response to extracellular and synaptic excitation of the cells populating the target structure (the neural response computation problem). The volume conduction problem can be formulated using dedicated software like COMSOL and solved through finite element modeling, as it is presented in Joarder et al.^80^ The neural response computation problem should exploit both components shown in Cottaris and Elfar^81^ for the retinal network modeling, and Meng et al.^82^ for biophysically accurate morphological models of RGCs. The driving action on each computational node across the RGC morphology would thus include synaptic excitation from other cells in the retinal network and the extracellular potential computed through volume conduction. While point-neuron models are currently the preferred choice for modeling other retinal cells like bipolar and amacrine cells, morphologically complex models of such cells may be developed in the future, including more complex spatial interactions between them and RGCs.

Biophysical models of RS would also allow the determination of the elongation of the phosphenes generated by the stimulation of passing fibers, which would contribute together with the elongation caused by the mismatch in the distributions of receptive fields and RGC somas to the distortion of the percepts produced through RS. Having neglected this well-known phenomenon provides thus a best-case scenario on the quality of the perceptions elicited by RS, which have then been compared to ONS. While we have determined through biophysical modeling of ONS that the presented geometrical method produces admissible patterns of activation even though it completely abstracted from any biophysical simulation and even from the determination of the optimal stimulation protocol, the same thing should be determined for RS.

Finally, an important limitation of the present study is that much of the complexity in the response of RGCs to electrical stimulation has been neglected. RGCs can be divided into several functional groups based on their receptive field shape and properties.^83^ The response to electrical stimulation of each of these families is only marginally characterized,^84^^,^^85^ and the understanding of their contribution to the formation of visual percepts is not yet satisfactory. Since they are arranged in superimposed mosaics, selective characterization of different RGC types in stimulation is extremely challenging. Future works should add more detail on the modeling of distinct families of RGCs both at the level of the retina and of the optic nerve, which could have an impact on the produced visual perceptions.

While much work is still needed to produce biophysical models capturing the complexity of the human retinal network and visual responses, simpler phenomenological models, like the one introduced in the present work, will play an important role in the development and characterization of novel neuroprosthetic devices. Moreover, many of the described limitations are expected to similarly affect the modeling of the responses to RS and ONS, and thus may not introduce substantial bias in the comparison of such technologies through computational models.

## Resource availability

### Lead contact

Further information and requests should be directed to the lead contact, S.R. (romeni.simone@hsr.it).

### Materials availability

This study did not generate new unique reagents.

### Data and code availability

• The dataset of visual stimuli employed in our psychophysical experiments as well as the anonymized subject responses are available in GitHub, at the link https://github.com/s-romeni/optic_nerve_stim__geometrical_model.
• All original code can be found in GitHub, at the link https://github.com/s-romeni/optic_nerve_stim__geometrical_model.
• Any additional information required to reanalyze the data reported in this paper is available from the lead contact upon request.

## Acknowledgments

This work was partly funded by the 10.13039/100009152Bertarelli Foundation; #NEXTGENERATIONEU (NGEU) and funded by the Ministry of University and Research (MUR); National Recovery and Resilience Plan (NRRP); projects MNESYS (PE0000006) – A Multiscale integrated approach to the study of the nervous system in health and disease (DN. 1553 11.10.2022); THE (IECS00000017) - Tuscany Health Ecosystem (DN. 1553 11.10.2022); and Association Sulle Ali Di Un Sogno ONLUS.

## Author contributions

Conceptualization, S.R. and S.Micera; Methodology, S.R., D.D.L., L.P., and S.Moccia; Software, S.R., D.D.L., L.P., G.M., and L.T.; Validation, S.R., D.D.L., and L.P.; Formal Analysis, S.R. and L.P.; Investigation, D.D.L. and L.P.; Resources, S.Micera; Writing – Original Draft, S.R., L.P., and S.Micera; Writing – Review and Editing, S.R., L.P., D.D.L., and S.Micera; Visualization, D.D.L. and L.P.; Supervision, S.Moccia and S.M.

## Declaration of interests

The authors declare no competing interests.

## STAR★Methods

### Key resources table

**Table:** 

REAGENT or RESOURCE	SOURCE	IDENTIFIER
**Deposited data**		

Reconstructed perception images	This paper	https://github.com/s-romeni/optic_nerve_stim__geometrical_model
Experimental data from psychophysical experiment	This paper	https://github.com/s-romeni/optic_nerve_stim__geometrical_model

**Software and algorithms**		

Python	Python Software Foundation	https://www.python.org
NEURON Simulation Environment	Duke, Yale, and the BlueBrain Project	http://neuron.yale.edu/neuron/
Mask R-CNN	Matterport	https://github.com/matterport/Mask_RCNN
Geometrical optimization of optic nerve and retinal stimulation	This paper	https://github.com/s-romeni/optic_nerve_stim__geometrical_model

### Experimental model and study participant details

Ten subjects (3 females, 25 ± 2 years old, western European) with normal or corrected-to-normal vision volunteered for our SPV experiment (see method details, SPV experiment). The Ethical Committee of Scuola Superiore Sant’Anna approved this study (study approval number 7/2023). All participants expressed their informed consent.

### Method details

#### Spaces and systems of reference

Each modeled RGC was characterized by

• the location $\left(\rho_{\text{RF}}^{i},\theta_{\text{RF}}^{i}\right)$ and the radius $\sigma_{\text{RF}}^{i}$ of its receptive field in the visual field;
• the location of its soma $\left(\rho_{\text{soma}}^{i},\theta_{\text{soma}}^{i}\right)$ in the frame of reference of the retina;
• the location of its axon/fiber $\left(\rho_{\text{soma}}^{i},\theta_{\text{soma}}^{i}\right)$ in the section of the optic nerve.

In order to follow the generation of these coordinates one from the other, we will start from the fiber coordinates, and then compute the soma coordinates and the receptive field parameters.

##### RGC axons in the optic nerve

We defined the optic nerve section as a circle centered in the origin with radius $R_{\text{ON}}=2.5$ mm. We sampled 10,000 pairs of uniformly distributed variables $\left(u_{\rho }^{i},u_{\theta }^{i}\right)$ in (0, 1), and we obtained uniformly distributed locations for ON fibers (RGC axons) in the ON section via the map

$$\left\{ \begin{array}{c} \theta_{\text{fiber}}^{i}=2\pi \cdot u_{\theta }^{i}\\ \rho_{\text{fiber}}^{i}=R_{\text{ON}}\cdot \sqrt{u_{\rho }^{i}} \end{array}\right.$$

##### RGC somas in the retina

The retina reference frame was centered in the fovea. The presented model corresponds to the left eye. The positive x direction corresponded to proceeding nasally while the positive y direction to proceeding superiorly. We defined a map to obtain the locations of the RGC somas in the retina using 2D inverse transform sampling. We started from the density $d^{*}\left(\rho ,\theta \right)$ of RGC somas in the human retina reported in^46^ and corrected it to a new density d(ρ,θ) so that an ellipse centered 4 mm nasal to the fovea with vertical axis length 1.77 mm and horizontal axis length 1.88 mm corresponding to the optic disc was voided of RGC somas.

We then defined a probability density function (PDF) $f_{\rho ,\theta }\left(\rho ,\theta \right)$ normalizing d(ρ,θ), or

$$f_{\rho ,\theta }\left(\rho ,\theta \right)=\frac{d\left(\rho ,\theta \right)}{\iint d\left(\rho ,\theta \right)\;\rho \;d\rho \;d\theta }$$

By marginalization, we can obtain the PDF of the variable θ:

$$f_{\theta }\left(\theta \right)=\int f_{\rho ,\theta }\left(\rho ,\theta \right)\rho \;d\rho$$

Using the definition of cumulative distribution function (CDF), we can obtain

$$F_{\theta }\left(\theta^{\prime }\right)=\int_{0}^{\theta^{\prime }}f_{\theta }\left(\theta \right)\;d\theta$$

and using Bayes rule and the CDF definition we finally obtain the conditional CDF

$$F_{\rho |\left(\theta =\theta^{\prime }\right)}\left(\rho^{\prime }\right)=\frac{\int_{0}^{\rho^{\prime }}f_{\rho ,\theta }\left(\rho ,\theta^{\prime }\right)\rho \;d\rho }{f_{\theta }\left(\theta^{\prime }\right)}$$

The 2D inverse transform sampling employs the map

$$\left\{ \begin{array}{c} \theta_{\text{soma}}^{i}=F_{\theta }^{-1}\left(u_{\theta }^{i}\right)\\ \rho_{\text{soma}}^{i}=F_{\rho |\left(\theta =\theta_{\text{soma}}^{i}\right)}^{-1}\left(u_{\rho }^{i}\right) \end{array}\right.$$

to obtain RGC soma locations distributed according to the PDF $f_{\rho ,\theta }\left(\rho ,\theta \right)$.

##### RGC receptive fields in the visual field

From Watson,^47^ we took the cumulative count functions ${\text{CC}}_{\text{soma}}\left(\rho^{\prime },\theta^{\prime }\right)$ and ${\text{CC}}_{\text{RF}}\left(\rho^{\prime },\theta^{\prime }\right)$ determining the number of somas and receptive fields with $\theta =\theta^{\prime }$, and $\rho <\rho^{\prime }$, respectively. The location $\left(\rho_{\text{RF}}^{i},\theta_{\text{RF}}^{i}\right)$ of the receptive field is obtained from $\left(\rho_{\text{soma}}^{i},\theta_{\text{soma}}^{i}\right)$ by setting $\theta_{\text{RF}}^{i}=\theta_{\text{soma}}^{i}\equiv \theta^{i}$, and $\rho_{\text{RF}}^{i}$ as the value $\rho^{i}$ which solves ${\text{CC}}_{\text{RF}}\left(\rho^{i},\theta^{i}\right)={\text{CC}}_{\text{soma}}\left(\rho_{\text{soma}}^{i},\theta^{i}\right)$.

Finally, the radius of the receptive field of the cell $\sigma_{\text{RF}}^{i}$ was obtained from its eccentricity $\rho_{\text{RF}}^{i}$ through the linear relation presented in Watson.^47^

##### Generalization of our geometrical model to different species

We show the generalization of the retinotopic map of assignment to pigs (*Sus scrofa*) and macaques (*Macaca mulatta*) using the empirical densities of the RGC somas in the retina found in Garcá et al.^51^ and Perry et al.,^52^ respectively. The empirical density in pig retina has approximately the same shape that in the rabbit (New Zealand red rabbit) retina,^86^ for which we could not find explicit data. In both cases, we assumed that $\left(\rho_{\text{soma}}^{i},\theta_{\text{soma}}^{i}\right)=\left(\rho_{\text{RF}}^{i},\theta_{\text{RF}}^{i}\right)$ since we did not find any available measurement of ${\text{CC}}_{\text{soma}}\left(\rho^{\prime },\theta^{\prime }\right)$ and ${\text{CC}}_{\text{RF}}\left(\rho^{\prime },\theta^{\prime }\right)$.

##### Inhomogeneous density of RGC axons in the optic nerve

We started from the density $d_{\text{fiber}}\left(\rho ,\theta \right)$ of RGC axons in the human optic nerve reported in^53^ and substituted

$$\left\{ \begin{array}{c} \theta_{\text{fiber}}^{i}=2\pi \cdot u_{\theta }^{i}\\ \rho_{\text{fiber}}^{i}=R_{\text{ON}}\cdot \sqrt{u_{\rho }^{i}} \end{array}\right.$$

with

$$\left\{ \begin{array}{c} \theta_{\text{fiber}}^{i}=F_{\theta }^{-1}\left(u_{\theta }^{i}\right)\\ \rho_{\text{fiber}}^{i}=F_{\rho |\left(\theta =\theta_{\text{fiber}}^{i}\right)}^{-1}\left(u_{\rho }^{i}\right) \end{array}\right.$$

according to the 2D inverse transform sampling formulas presented above for the retina.

#### Generation of the visual stimuli

##### Static visual stimuli

We took 90 pictures with an RGB-D Intel RealSense Depth Camera D455 in three settings, namely simulating the point of view of a subject sitting in front of a table, standing in an indoor environment and in an outdoor one (30 pictures for each setting). For each visual scene, the camera captured an RGB image with a 90° × 65° field of view (FOV), and a depth map with an 87° × 58° FOV. The depth map range was between 0.3 m and 20 m. The size of both the RGB pictures and the depth maps was 480 × 640 pixels, but since the FOVs of the two images were different, we manually aligned the images to fit a 480 × 640 pixel, 90° × 65° FOV map. Depth maps were processed with a hole-filling filterto recorver missing information.

The RGB images underwent semantic segmentation through a convolutional neural network (CNN) model called Mask R-CNN,^87^ whose implementation is available at the following link: https://github.com/matterport/Mask_RCNN. The available network was trained on the COCO dataset,^88^ comprising 91 object labels, including persons, cars, chairs, cups, and other objects of common use in daily life. The experiments were conducted on an HP Pavilion Laptop 15-eg0xxx, 11th Gen Intel(R) Core(TM) i7-1165G7 processor, 2.80 GHz CPU, NVIDIA GeForce MX450 GPU.

Semantic segmentation produced an image containing a pixel-wise classification of the input images: each pixel has a different value depending on the object it belongs to (e.g., person, car, etc.). The set of pixels belonging to a same object constitutes a mask. The application of the Mark R-CNN to the RGB images thus resulted in the determination of a set of masks for each visual scene.

Each mask was then associated to a gray level $I_{i}$, and displayed on a black background in the finally obtained visual stimuli. The gray levels were associated to each mask by taking as reference a certain range of depths, where the minimum depth $d_{\min}$ corresponded to white ($I_{i}=1$) and the maximum depth $d_{\max}$ to black ($I_{i}=0$). These ranges were chosen accordingly to the scenarios. For the pictures taken in front of a table we chose a range $\left(d_{\min},d_{\max}\right)$ of 0.3 m–1.5 m, for the indoor scenario we chose a range of 0.5 m - 5m and for an outdoor scenario we chose a range of 1.5 m–16 m. For table and indoor scenes, the gray level was associated to the masks of the objects using a linear function:

$$I_{i}=\frac{d_{\min}-d_{i}}{d_{\max}-d_{\min}}+1$$

Where $I_{i}$ and $d_{i}$ correspond to the gray and depth of the i-th instance, and $d_{\min}$ and $d_{\max}$ are the extremes of the range distance. For outdoor scenes, the gray level was assigned as follows:

$$I_{i}=a\cdot d_{i}^{2\;}+b$$

with:

$$a=\frac{1}{d_{\min}^{2}-d_{\max}^{2}},b=-\frac{d_{\max}^{2}}{d_{\min}^{2}-d_{\max}^{2}}$$

##### Dynamic visual stimuli

The dynamic visual stimuli consisted in videos of the duration of 3 s displaying blocks moving across the screen in the horizontal direction. Each short video contains a different number of randomly generated blocks of different sizes and shades of gray, which simulate objects moving at different velocities and distances from the subject. In total, 30 videos were generated.

#### Conversion between images and firing rate patterns

Here, the FR of each RGC is a static linear function of the applied visual stimulus, through a bidimensional sensitivity function with the shape of a Gaussian function in the image space. This is a simplification of classical center-surround linear filter model of RGC response introduced in,^89^^,^^90^ without the smaller surround response, or a linearization of the model presented in.^71^ From a computational point of view, each cell is associated to a grayscale RF image ${\text{RF}}_{i}$ with the same size as the proposed visual scenes containing a homogeneous bivariate Gaussian function with center $\left(\rho_{\text{RF}}^{i},\theta_{\text{RF}}^{i}\right)$ and bandwith $\sigma_{\text{RF}}^{i}$ depending linearly upon the eccentricity $\rho_{\text{RF}}^{i}$ as discussed in the previous section. The firing rate of the i-th RGC is obtained through the image convolution (element-wise multiplication):

$${\text{FR}}_{i}\left(\text{visual}\;\text{scene}\right)=\text{visual}\;\text{scene}\;⨂\;{\text{RF}}_{i}$$

The scaling of the Gaussian in ${\text{RF}}_{i}$ is such that when applied to a white image it produces a 300 Hz firing rate. The maximum imposed firing rate of 300 Hz was obtained as the maximum firing rate when stimulating our biophysical model of optic nerve fibers (see the corresponding section of the Methods below) up to 300 Hz like in.^4^

#### Distortion of the visual scenes according to RS and ONS

Subdivisions into regular grids of the retina and optic nerve represented ideal implant electrode arrays. For RS, we employed electrode spacing and positioning of the Argus II electrode array, defining the side of a cell as the interelectrode distance of the array. For ONS, we covered the square circumscribed to the optic nerve section with electrode arrays with number of contacts equal to 10 × 10, 14 × 14, 32 × 32. Additionally, we produced an electrode array obtained from the 14 × 14 arrangement by introducing an internal 4 × 4 grid of larger grid cells where each large grid cell is composed by a 4 × 4 arrangement of smaller grid cells.

The natural FR for each RGC has been computed for each visual scene according to the formalism presented in last section. Given an electrode cell, the FR imposed by stimulation to all the RGCs in the cell is set to the maximum FR in the cell if at least 30% of the RGC in the cell are active (FR > 0), and zero otherwise. It is important to remark that this encoding heuristic does not need to be biologically grounded, rather it is established so that the reconstructed perceptions resemble as much as possible to the intended visual scenes.

The visual scenes reconstructed from the firing rates were obtained through a linear combination of the RF images with the cell FRs, namely

$$\text{perception}\propto \sum_{i}{\text{FR}}_{i}\cdot {\text{RF}}_{i}$$

#### SPV experiment

##### Experimental setup

The experiment consisted in the presentation of the SPV images and videos on a 75 inches LED screen. The subjects were asked to sit in front of the screen, at an 80 cm eye-to-screen distance, so that the screen occupied a field of view of 90° × 60°, corresponding to the Intel RealSense D455 field of view. They were also asked to constantly keep their gaze on a red fixation point, visible throughout the whole experimental session on the center of the screen. They could explore the scene and simulate gaze shifting by using the arrows on a keyboard at their disposal.

##### Visual stimuli presentation

The experiment was composed of three tasks. During the first two, static stimuli were presented, while the third encompassed the presentation of dynamic visual scenes. Task 1 and task 2 involved 45 images each. Of these, 15 were undistorted, 15 were distorted simulating ON prosthetic vision, and 15 were distorted simulating retinal prosthetic vision. Each of these 3 image groups comprised 5 images for each among the table, indoor and outdoor scenarios. In both task 1 and task 2, the first image group to be delivered to the subjects was the undistorted, so that they could familiarize themselves with the visual stimuli. The optic nerve and retinal prosthetic vision groups of images followed in random order. Images belonging to the same scenario were displayed subsequently, but the presentation order of the different scenarios was random. Task 3 consisted in the presentation of the 30 short videos, which were divided into series of 10 videos each. Again, a series of videos was undistorted while the other two were distorted simulating optic nerve and retinal prosthetic vision, respectively. The first series of videos delivered to the subjects comprised undistorted videos, to allow them to familiarize themselves with the visual stimuli. The other two series were displayed in random order. The presentation order of the images and videos was randomized across subjects, meaning that the same tasks did not necessarily involve the same visual stimuli, and that the images were delivered to different subjects with different distortions.

##### Task description

In task 1, subjects were required to count the number of separate objects present in the scene, to sort them by distance (increasing distance from the subject), and by size (from largest to smallest).

In task 2, we asked subjects to classify an object indicated by the experimenter in the proposed visual scenes choosing one of three possible labels. The proposed labels corresponded to objects likely to be found in the corresponding scenario. For example, in the table scenario, we could provide labels like “cup”, “glass”, or “bottle” but not labels as “table”, “chair”, “car”, or “bike”.

In task 3, we asked subjects to count the number of objects appearing on screen during each proposed video.

##### Random experiments

In order to establish whether simulated ONS and RS provided functional visual perceptions, we evaluated the performances that would have been obtained for our tasks if the subjects answered in a completely random way. We thus ran the same experiments run for our real subjects on the same number of “virtual subjects”, whose answers were determined as follows. For task 1, we computed the proportion of images from our dataset of 90 visual scenes where N objects were present and then we sampled for each response given by each virtual a number $N^{\prime }$ according to the above distribution. This should simulate the prior knowledge of the subjects that understand while doing the experiment that the number of stimuli can vary across a wide range but that each possibility has different probabilities of occurrence. For distance and dimension ordering, we sampled a random permutation of the numbers from 1 to M, where M is the actual number of objects in the scenes. For task 2, we sampled a random number between 1 and 3 to represent the target class, and another independent number to represent the answered class. For task 3, we proceeded in the same way as for the object counting in task 1. We then computed the same quantities computed for real subjects and obtained the first, second, and third quartiles of the obtained distributions for virtual subjects.

##### Task performance

In tasks 1 and 3, absolute errors and relative errors in object count were computed as

$$\varepsilon_{A}=\left|n_{\text{true}}-n\right|,\varepsilon_{R}=\frac{\left|n_{\text{true}}-n\right|}{n_{\text{true}}}$$

respectively, where $n_{\text{true}}$ is the number of objects actually present in the scene and n the number of objects identified by the subject.

In task 1, the ability of the subjects to order object distances and sizes was measured using Kendall’s rank correlation coefficient (often abbreviated Kendall’s τ) between two arrays containing the indicated object orderings through ordinal numerals, in its implementation available in scipy.stats.kendalltau. The value of Kendall’s τ was only computed when the subject provided the correct number of objects was identified.

In task 2, the classification error was computed as the proportion of mislabeled samples among all the proposed samples.

In all cases, subject-wise performances are shown through their mean value and the interval between the 5^th^ and 95^th^ percentiles. The more classic use of median and 1^st^ and 3^rd^ quartiles was not regarded as particularly informative as in most cases the subjects provided the correct responses, thus pushing the error distributions toward zero.

##### Comparing different ONS electrode grids

We enrolled five further subjects (3 females, 26 ± 5 years old) and performed task 2 employing scenes reconstructed employing 100-, 196- and 1024-electrode grids in the optic nerve and reported object recognition accuracies.

#### ON fiber biophysical model

The biophysical model of the ON fibers has been taken from.^32^^,^^49^^,^^50^ We set the fiber diameter to 1 μm, the number of Ranvier node to 31, and we record in correspondence to the 26^th^ Ranvier node. From the time-course of the recorded potential, we found action potentials as peaks with height larger than 10 mV. The simulation is run for 1000 ms with a time step of 0.1 ms. Each electrode, located in the center of its electrode cell, injects an independent sinusoidal current with a given current amplitude and frequency

$$i_{j}\left(t\right)=A_{j}\;\sin\left(2\pi f_{j}\right)$$

Because we assume all media to be time-invariant, we can write the potential due to the injection of charge from the stimulating electrodes as

$$v\left(r,t\right)=\sum_{j=1}^{n_{\text{sites}}}l_{j}\left(r\right)\cdot i_{j}\left(t\right)$$

where $l_{j}\left(r\right)$ is computed assuming to be in an infinite, homogeneous, anisotropic medium with transverse electrical conductivity $\sigma_{x}=\sigma_{y}\equiv \sigma_{xy}$ and a longitudinal electrical conductivity $\sigma_{z}$, obtaining

$$l_{j}\left(r\right)={\left(4\pi \sqrt{\sigma_{xy}\sigma_{z}\left[{\left(x_{j}-x\right)}^{2}+{\left(y_{j}-y\right)}^{2}\right]+\sigma_{xy}^{2}{\left(z_{j}-z\right)}^{2}}\right)}^{-1}$$

##### Ion channel dynamics

$$I_{A}=g_{A}\cdot a^{3}\cdot b\cdot \left(V-E_{K}\right)$$

$$\alpha_{a}\left(V\right)=0.015\cdot \frac{V+90}{1-\exp\left(-\frac{V+90}{10}\right)\;}$$

$$\beta_{a}\left(V\right)=0.25\cdot \exp\left(-\frac{V+30}{10}\right)$$

$$\alpha_{b}\left(V\right)=0.04\cdot \exp\left(-\frac{V+65}{20}\right)$$

$$\beta_{b}=\frac{60}{1+\exp\left(-\frac{V+35}{10}\right)}$$

$${I_{Na}=g}_{Na}\cdot m^{3}\cdot h\cdot \left(V-E_{Na}\right)$$

$$\alpha_{m}=0.45\cdot \frac{V+37}{1-\exp\left(-\frac{V+37}{10}\right)}$$

$$\beta_{m}=15\cdot \exp\left(-\frac{V+62}{18}\right)$$

$$\alpha_{h}=0.16\cdot \exp\left(-\frac{V+67}{20}\right)$$

$$\beta_{h}=\frac{2.4}{1+\exp\left(-\frac{V+37}{10}\right)}$$

$$I_{P}=g_{P}\cdot p^{3}$$

$$\alpha_{p}=0.0151\cdot \frac{V+19}{1-\exp\left(-\frac{V+19}{10.2}\right)}$$

$$\beta_{p}=-0.000379\cdot \frac{V+26}{1-\exp\left(\frac{V+26}{10}\right)}$$

$$I_{L}=g_{l}\cdot \left(V-E_{L}\right)$$

#### Factors affecting the quality of the output perception

We clarify that the sections below illustrate the steps necessary to replicate the presented results. The interest of this set of simulations was to describe the degradation of the output perception when certain hypotheses in the original model were modified. We could not find physiological ranges for the employed quantities. We have discussed the interest of performing more in-depth analyses of the sort, and the experimental analyses leading to the determination of realistic values for such quantities in the discussion.

##### Local shuffle parameters

For each fiber i uniformly random selected to be swapped, the fiber j to be swapped with was chosen according to the probability:

$$p_{i,j}=\frac{w_{i,j}}{\sum_{j}w_{i,j}}\text{,}$$

Where $w_{i,j}$ represents the weight for the probability of swapping the i-th fiber with the j-th fiber, and it is defined as follows:

$$w_{i,j}=\exp\;\left(-\frac{d_{i,j}^{2}}{2\sigma^{2}}\right)$$

$d_{i,j}^{2}$ represents the distance between the i-th and the j-th fibers in the optic nerve section and σ is a parameter that we set to the be 1/5 of the average fiber-fiber distance in the optic nerve. The number of swaps was set to 1/5 of the total number of fibers (2,000 fibers out of 10,000).

##### Global shuffle parameters

The target for the global shuffling is the RGCs inside the square inscribed in the circle representing the border of the optic nerve section. The square is equally divided into a 6-by-6 matrix of squared units, from now on simply called units. For each swap, a unit is randomly selected to be swapped with another unit, according to the same probability function used for the local swap, using the units’ centers to compute the distances $d_{ij}$ (now i and j are the indexes for the units). We set sigma to be 1/5 of the average distances among all the pairs of units’ centers.

We limited the swaps to occur only among squared units of the same size to ensure that each of them had a uniform distribution of RGCs and the same expected number of cells. Therefore, the overall optic nerve distribution was still uniform after any number of swaps. The number of swaps was set to be the nearest integer to 1/5 of the number of units.

After these swaps, as for the case of the local shuffling, each fiber had the same corresponding bodies, receptive fields and receptive centers than before, but could be located in a position in the optic nerve that did not resemble the perfect original retinotopy.

##### Electrode contact malfunctioning

We set to zero the firing rates of the fibers in the grid cells of a proportion of 10% of the contact sites, simulating the inability to stimulate through the chosen electrodes. The malfunctioning sites have been chosen randomly among the sites in the nerve section.

##### Death of optic nerve fibers by degeneration

We identified as dead a number of optic nerve fibers, and set to zero their firing rate when computing the reconstructed perception. We describe three different scenarios where a proportion of half of the fibers are degenerated.

Scenario 1: Uniform degeneration

$$p_{\text{uniform}}\left(\rho \right)=k_{0}$$

Scenario 2: Foveal degeneration

$$p_{\text{foveal}}\left(\rho \right)=\frac{k_{1}}{1+\exp\;\left(\rho -0.35\;R_{\text{RET}}\right)}$$

Scenario 3: Peripheral degeneration

$$p_{\text{peripheral}}\left(\rho \right)=\frac{k_{2}}{1+\exp\;\left(0.35\;R_{\text{RET}\;}-\rho \right)}$$

The parameters $k_{0},k_{1},k_{2}$ have been set so that the total proportion of degenerated cells is 0.5.

### Quantification and statistical analysis

For each task of the SPV experiment, we collected all the values of performance metrics across all subjects and tested the differences between undistorted, RS and ONS scenarios throrugh Kruskal-Wallis test. When a significant difference among the three groups was obtained, post-hoc Dunn test with Bonferroni correction was employed to perform pairwise comparisons. When significant, we reported the *p*-values in Figure 7.
