# Supplementary material for: A computational model to design wide field-of-view optic nerve neuroprostheses
Source: iScience. 2024 Nov 5;27(12):111321. doi: 10.1016/j.isci.2024.111321 (PMC11612796; doi:10.1016/j.isci.2024.111321)
Supplement: Document S1. Figures S1–S15 and Tables S1–S4 [file mmc1.pdf]

iScience, Volume *27*

Supplemental information

A computational model to design

wide field-of-view optic nerve neuroprostheses

Simone Romeni, Daniela De Luca, Luca Pierantoni, Laura Toni, Gabriele Marino, Sara Moccia, and Silvestro Micera

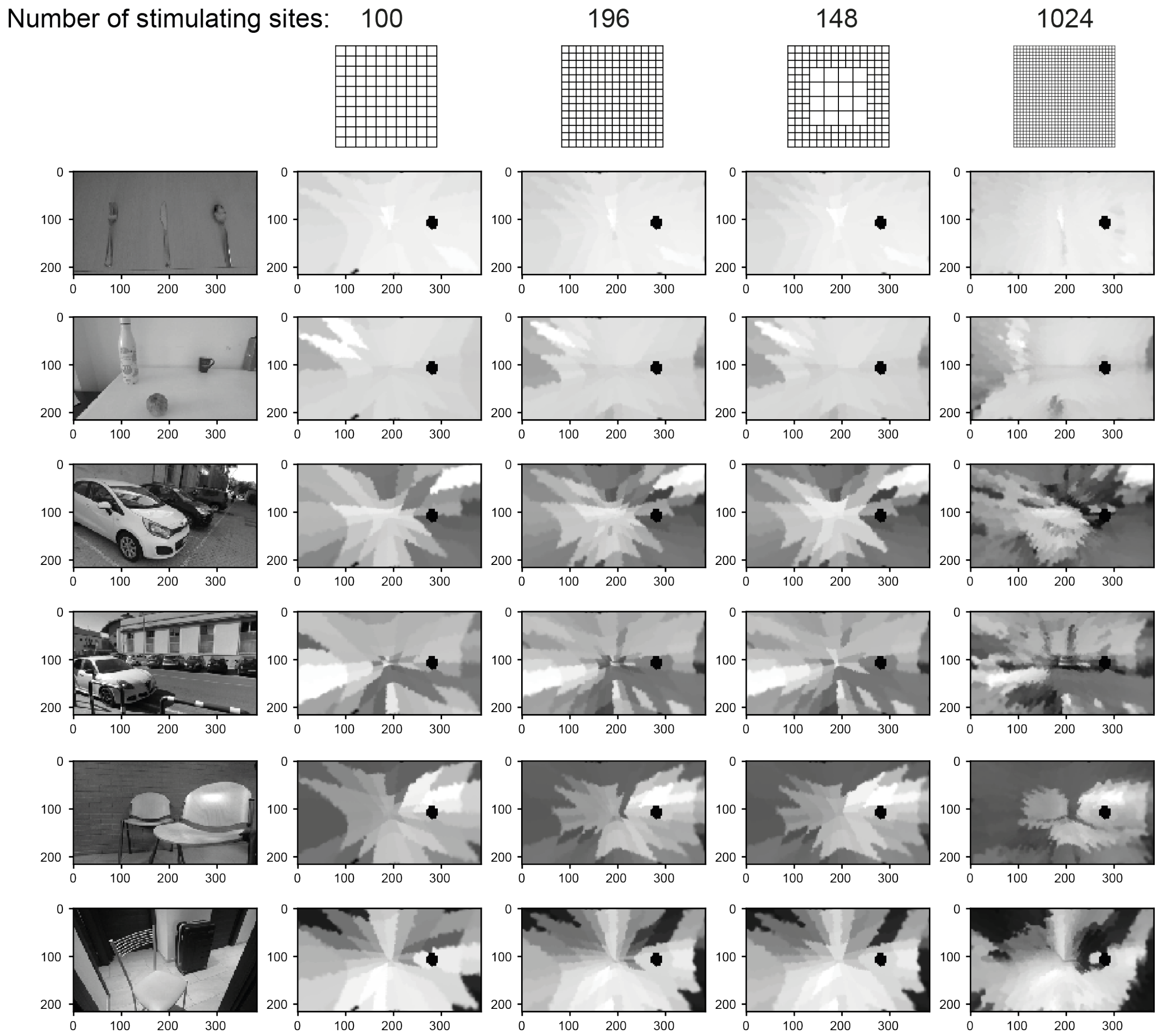

Supplementary Figure 1. ***Best achievable phosphene patterns for original visual scenes using different electrode grids,*** *related to* ***Figure 5***.

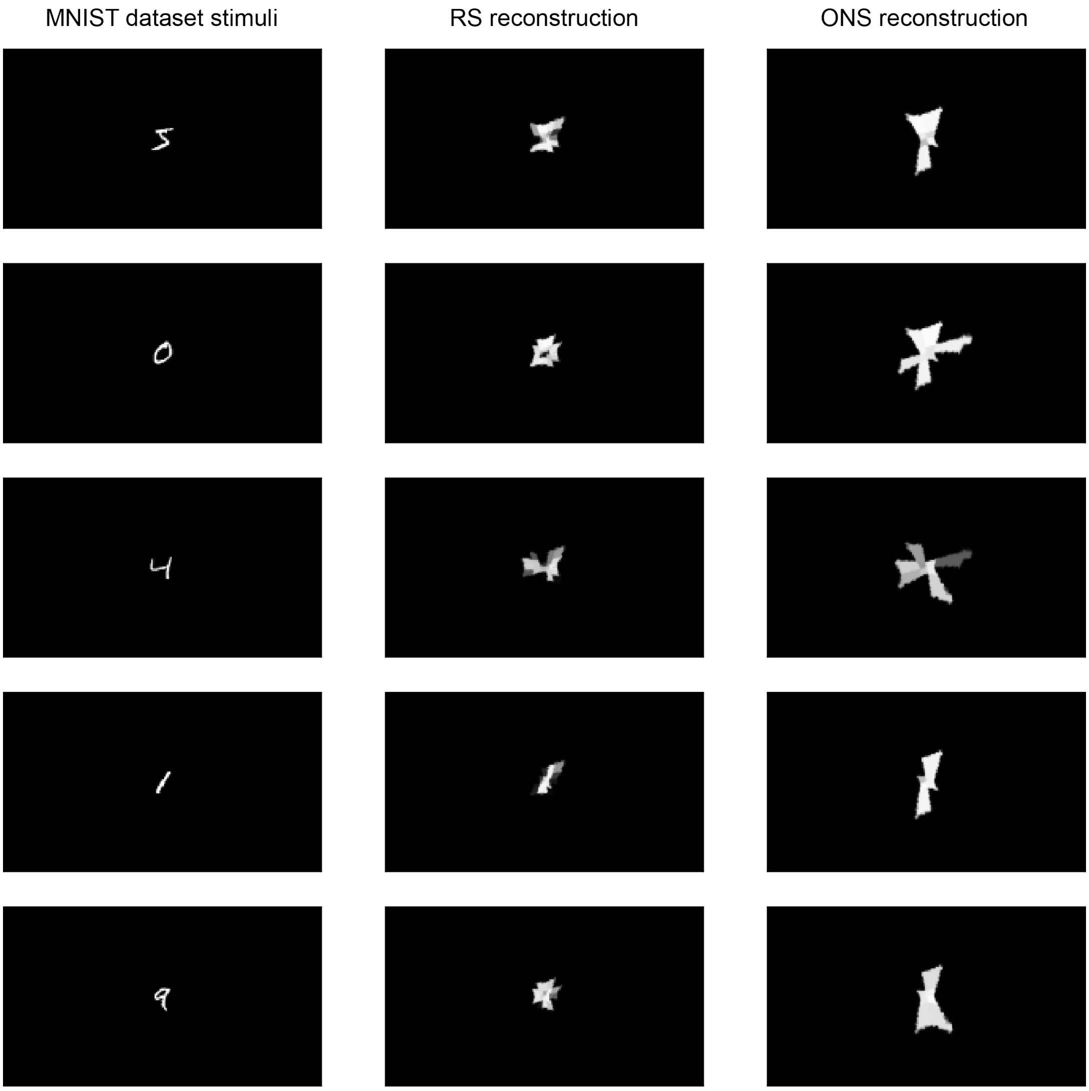

Supplementary Figure 2. ***Best achievable phosphene patterns for MNIST through retinal stimulation,*** *related to* ***Figure 5***. Best achievable phosphene patterns for MNIST ciphers located in the center of the visual field using our simulated retinal prosthetic vision and optic nerve prosthetic vision with 148 stimulating sites.

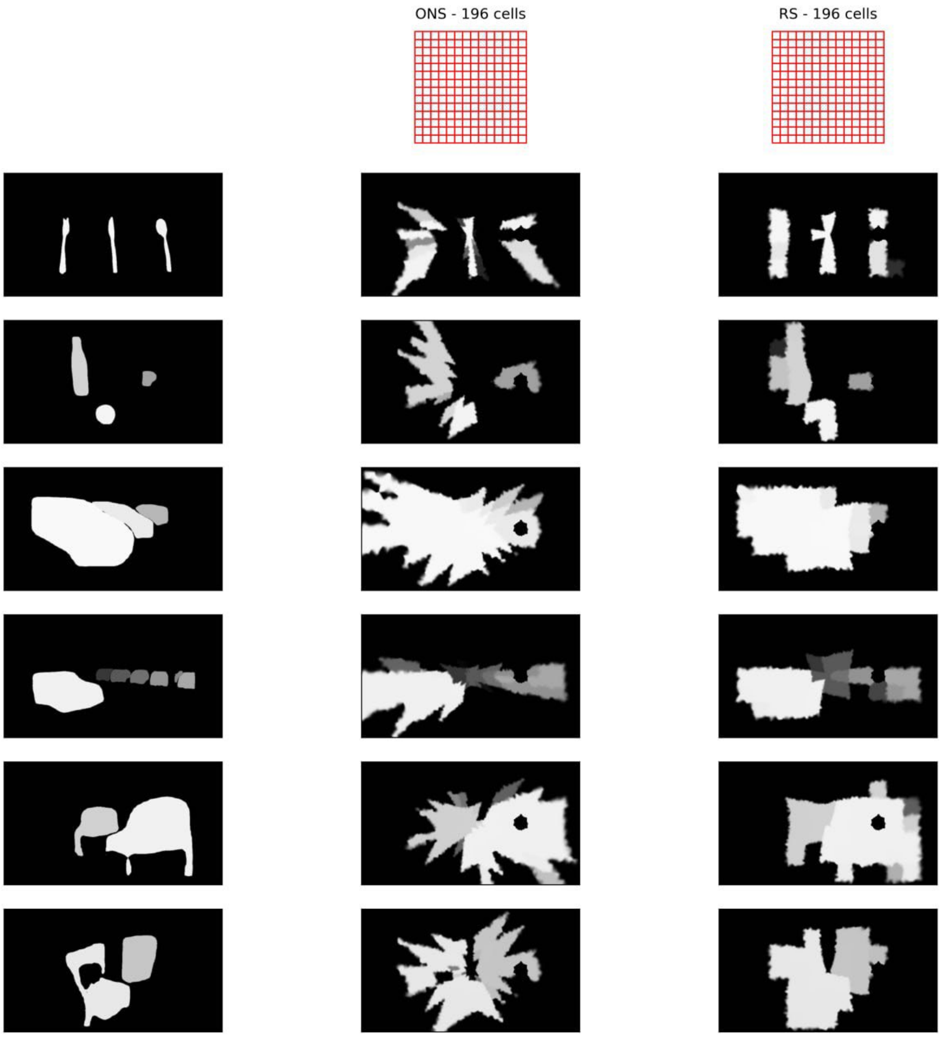

Supplementary Figure 3. ***Comparison between reconstructed visual scenes through ONS and RS employing 196 electrodes covering the whole optic nerve section or retinal surface****, related to* ***Figure 5****.*

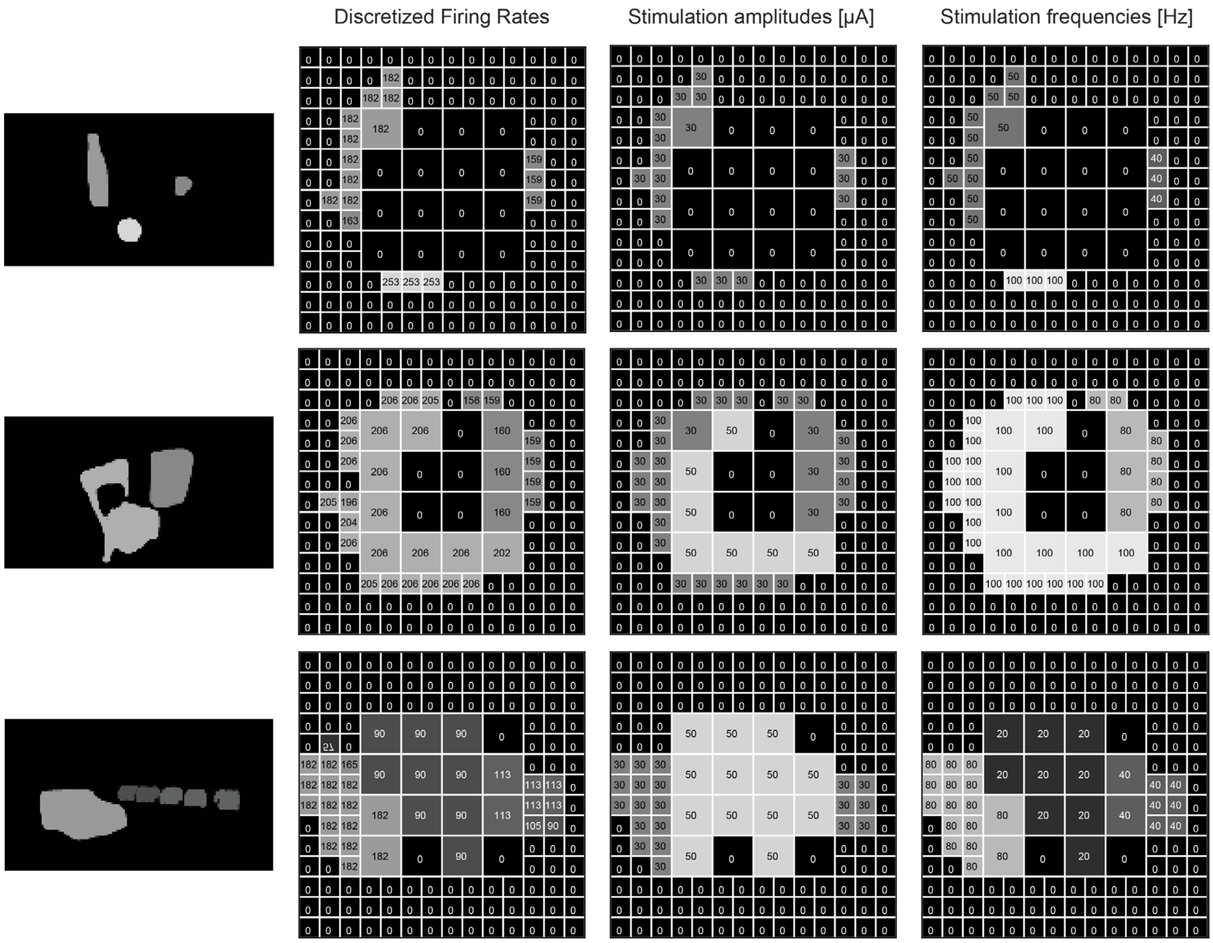

Supplementary Figure 4. ***Intermediate results for biophysical phosphene patterns***, *related to* ***Figure 6***. Discretized firing rates obtained from three visual scenes, stimulation amplitudes and frequencies that have been found through manual tuning to produce the phosphene patterns presented in **Figure 6**. All electrode arrays contain 148 stimulating sites.

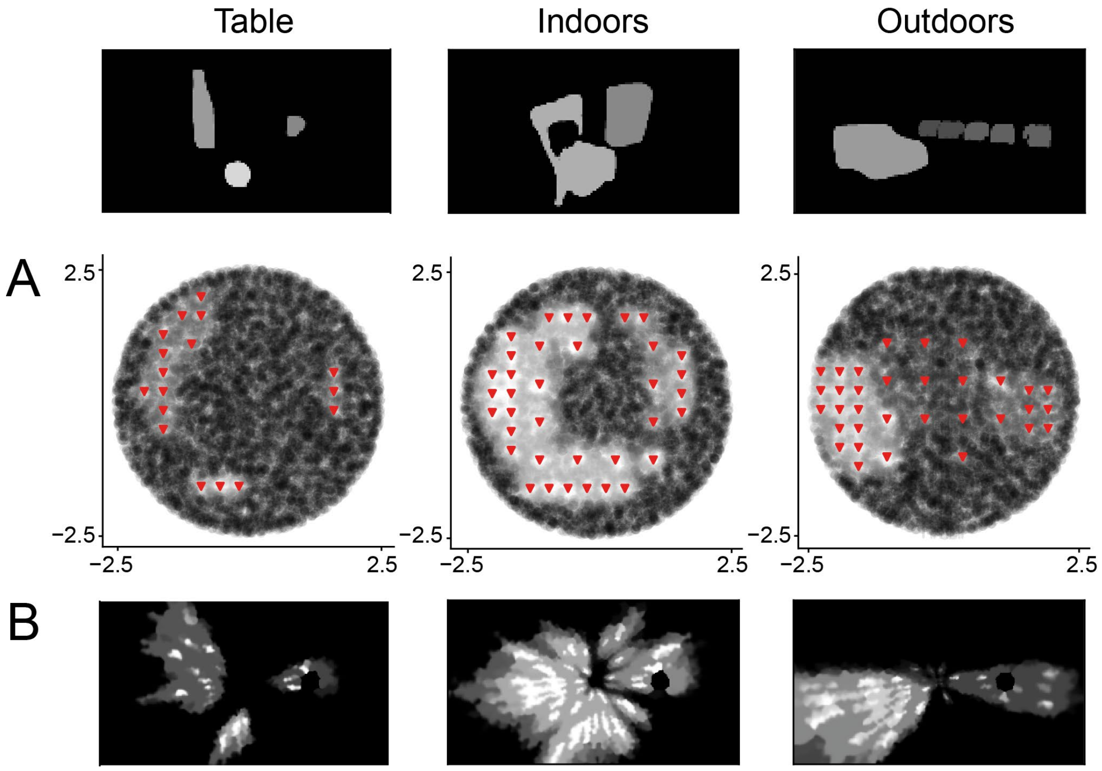

Supplementary Figure 5. ***Biophysical firing rates and phosphene patterns,*** *related to* ***Figure 6***. (**A**) Firing rate obtained in the optic nerve using the biophysical model to simulate a 500 ms stimulation on a subsampled population of 2,500 fibers. (**B**) Reconstruction of the stimuli based on the simulated firing rate on the subsampled population of 2,500 fibers.

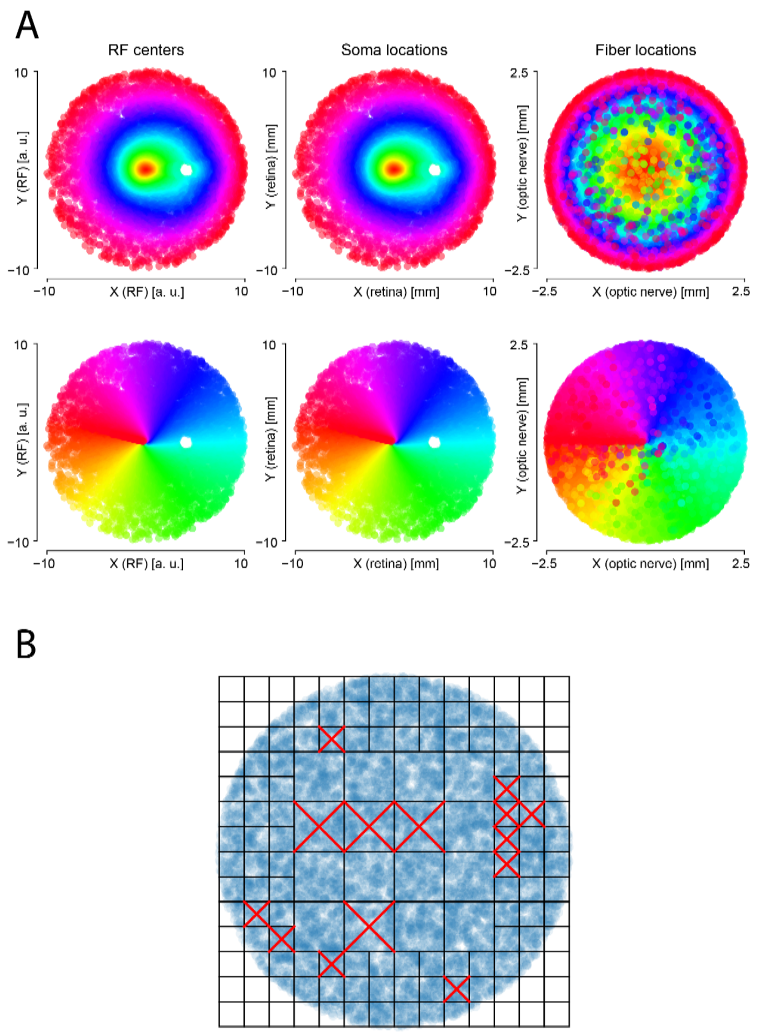

Supplementary Figure 6. *Imperfect retinotopy and electrode performance,* *related to* ***Figure 8*** (**A**) Radial and angular retinotopy maps for the introduced local shuffling. Each RGC is associated to a single color across visual field, retina and optic nerve section. (**B**) Layout of the electrodes that have been inactivated (whose grids have been marked with a red cross) to simulate electrode contact malfunctioning.

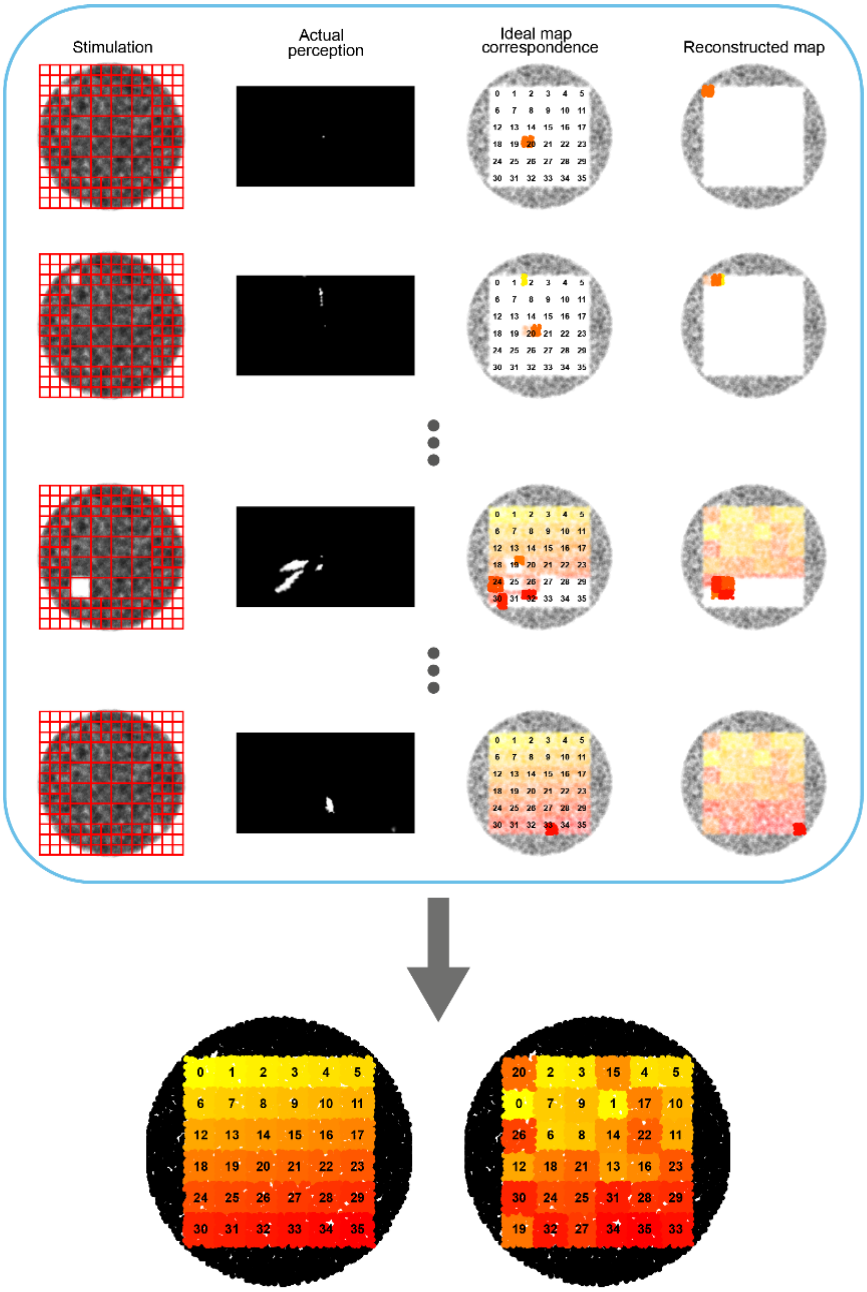

Supplementary Figure 7. Outline of the experimental procedure for the determination of the map of global shuffling, *related to **Figure 8***. The map of global shuffling displayed in the bottom of the figure provides a way to build a non-retinotopic model personalized on the target patient from the retinotopic geometrical model presented in this work. Each electrode in turn is activated, assuming the nerve to be retinotopic. The patient then refers phosphenes in a given part of the visual field, which corresponds to fibers in a different location with respect to the ones assumed to be stimulated if the nerve was retinotopic. Repeating for each available stimulating site, we manage to establish the map from the retinotopic geometrical model to the personalized non-retinotopic one.

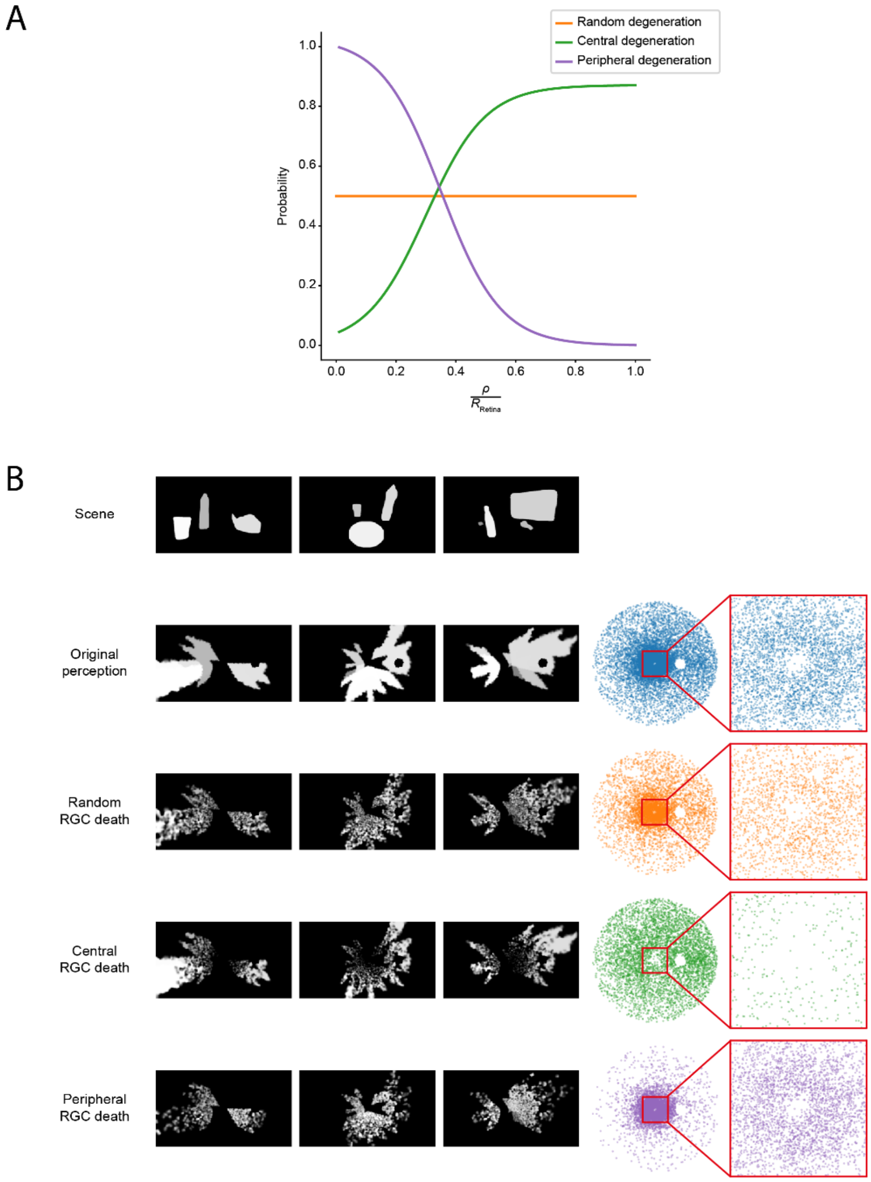

Supplementary Figure 8. ***Retinal ganglion cell death profiles***, *related to* ***Figure 8.*** (**A**) Profiles of imposed optic fibers degeneration with respect to retinal coordinates of their soma (degeneration originates at the level of the retina). The curves are tuned in such a way to inactivate in each case 50% of the RGCs. (**B**) Spatial pattern of cell degeneration and resulting perceptions for three visual scenes in the cases of uniform, foveal and peripheral degeneration.

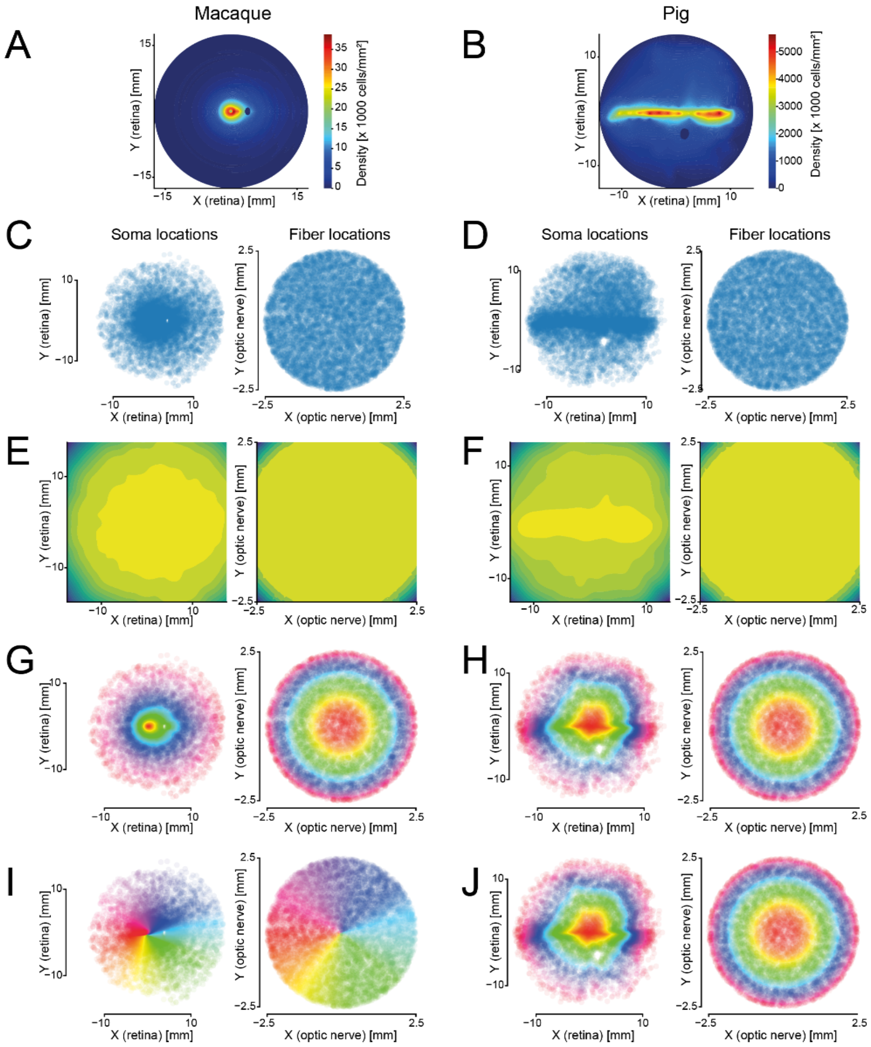

Supplementary Figure 9. ***Geometrical model for different species,*** *related to* ***Figure 2***. (**A-B**) Experimental densities of RGCs in the retina. (**C-D**) Scatterplots of RF center locations in the visual field, soma locations in the retina, and fiber locations in the optic nerve transverse section. (**E-F**) Contour plot of the density of RF centers, somas, and fiber locations; 10 levels are shown in all three cases. (**G-H**) Representation of radial retinotopy. Fibers of different colors correspond to different radial distances from the center of the optic nerve transverse section, the same color is used for each RGC. (**I-J**) Representation of angular retinotopy. Fibers of different colors correspond to different angular distances from the positive x-semiaxis of the optic nerve transverse section, the same color is used for each RGC. Panels on the left refer to macaque and panels on the right refer to pig anatomy.

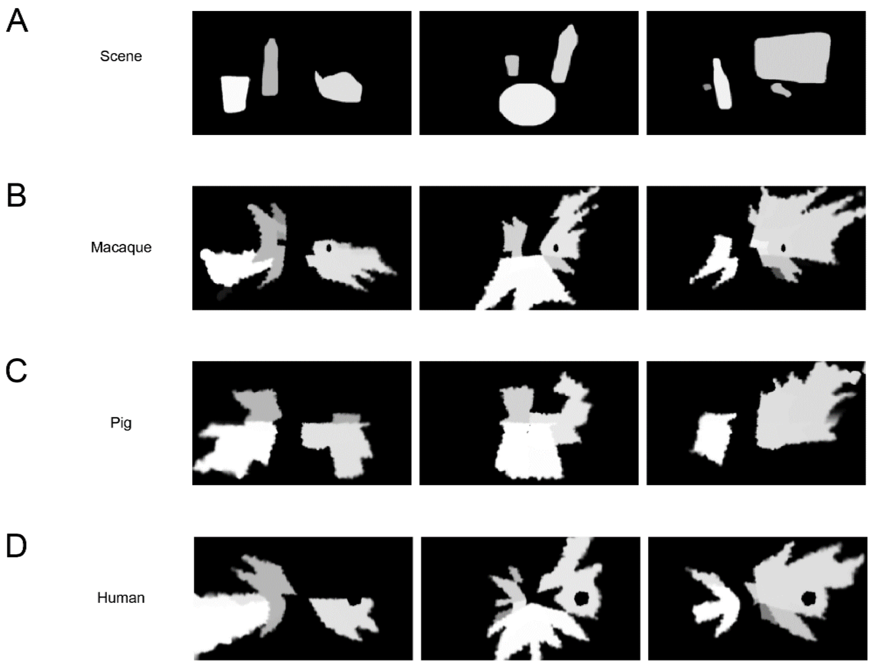

Supplementary Figure 10. **Original visual scene and best reconstructed scenes using our algorithm and geometrical models for macaque, pig / rabbit and human**, *related to **Figure 2** and **Figure 5***.

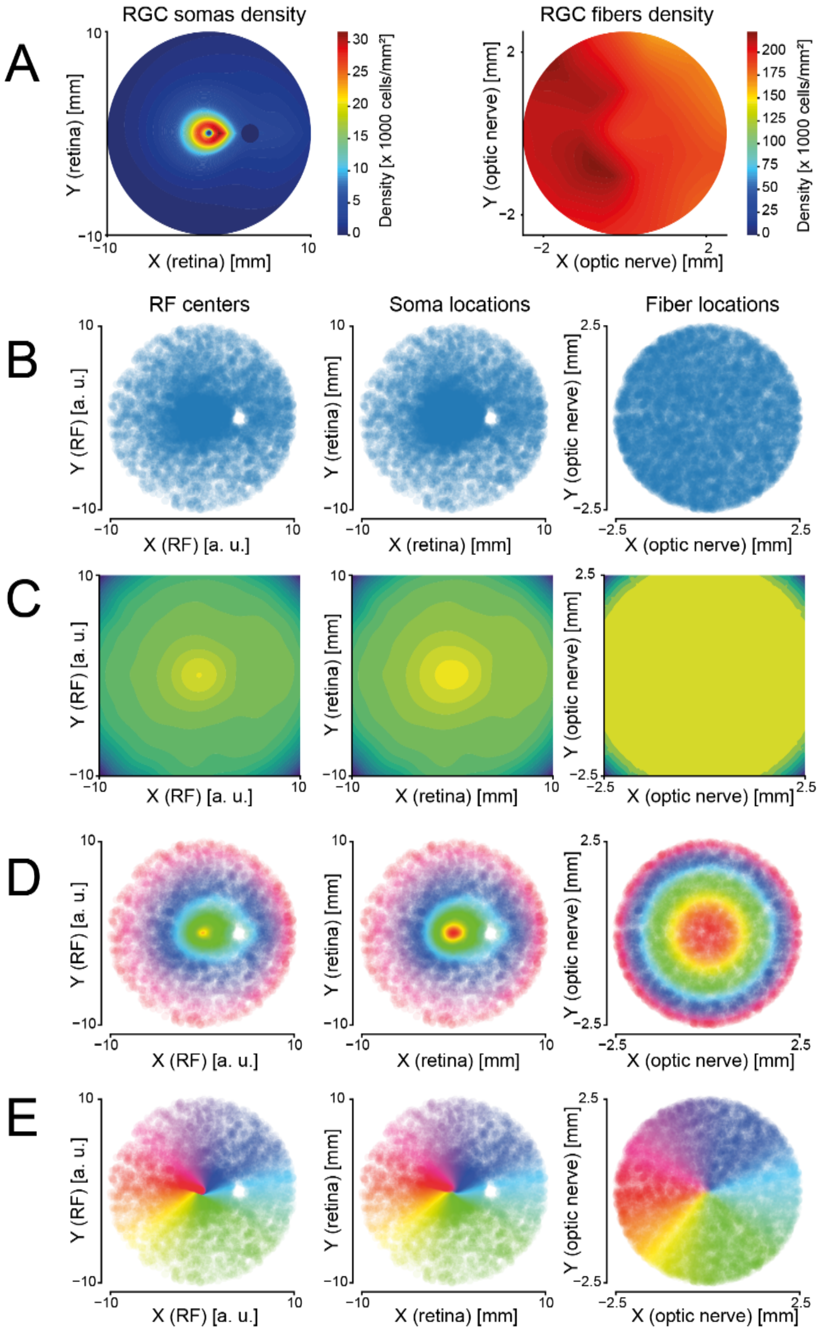

Supplementary Figure 11. ***Geometrical model for inhomogeneous optic nerve fiber distribution***, *related to* ***Figure 2***. (**A**) Experimental densities of RGCs in the retina and in the optic nerve section. (**B**) Scatterplots of RF center locations in the visual field, soma locations in the retina, and fiber locations in the optic nerve transverse section. (**C**) Contour plot of the density of RF centers, somas, and fiber locations; 10 levels are shown in all three cases. (**D**) Representation of radial retinotopy. Fibers of different colors correspond to different radial distances from the center of the optic nerve transverse section, the same color is used for each RGC. (**E**) Representation of angular retinotopy. Fibers of different colors correspond to different angular distances from the positive x-semiaxis of the optic nerve transverse section.

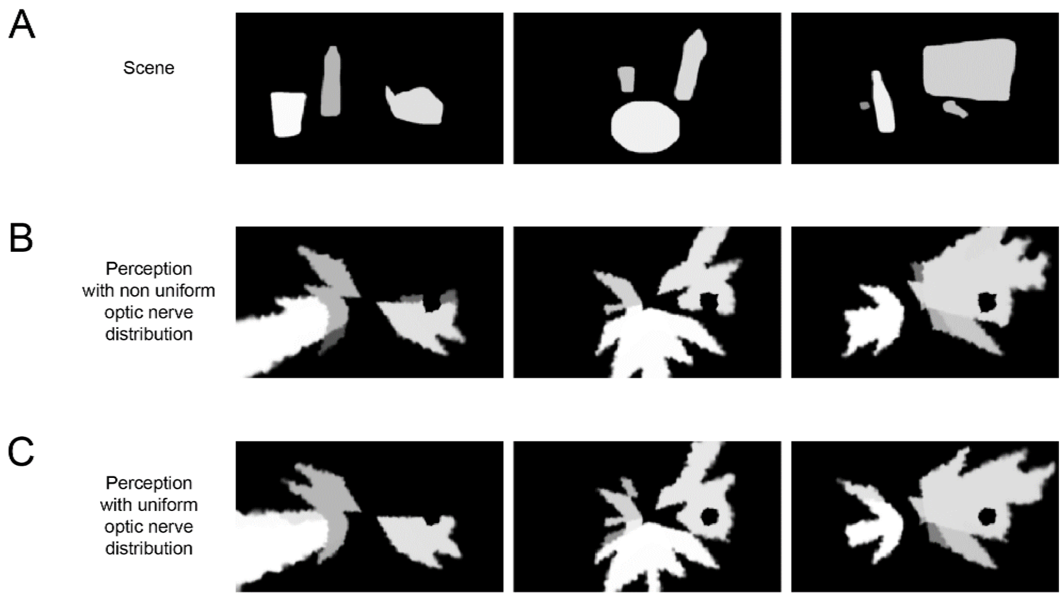

Supplementary Figure 12. **Original visual scenes and reconstructed visual perceptions with uniform and non-uniform density of optic nerve fibers***, related to* ***Figure 2***.

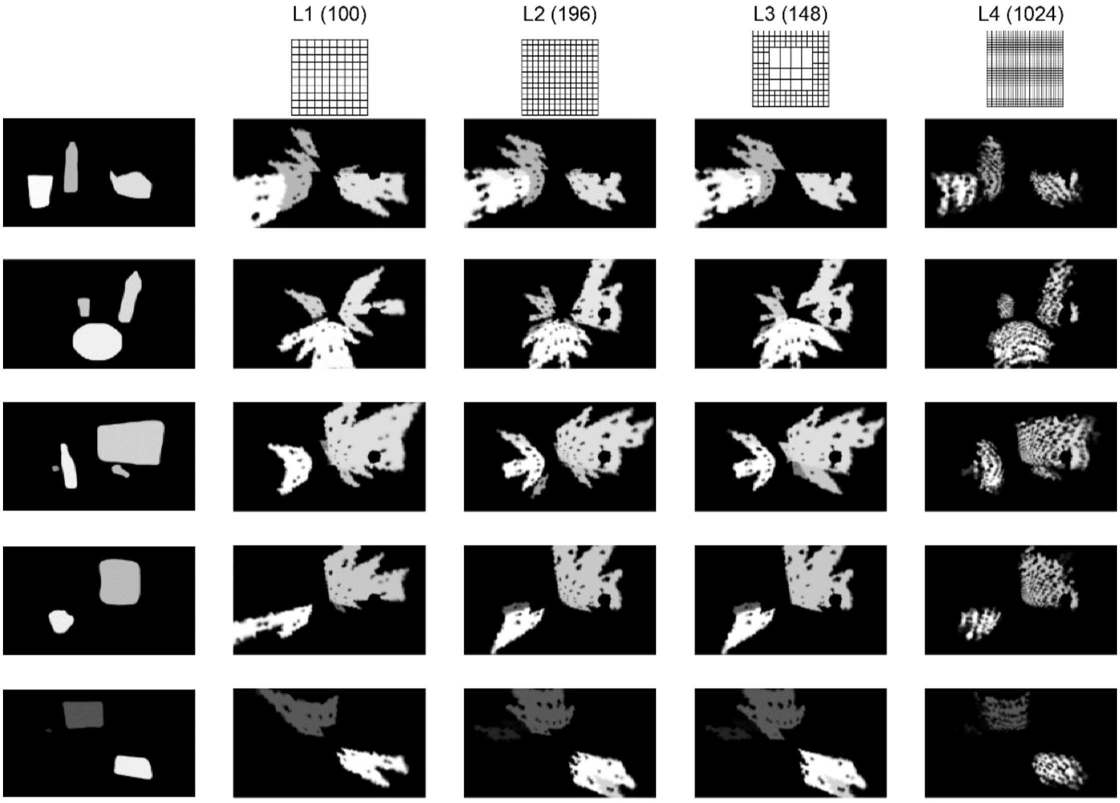

Supplementary Figure 13. **Cell death first scenario**, *related to **Figure 8***. Reconstructed perceptions supposing the death of the fibers closer than 50 µm from each implanted stimulating site for the different tested electrode array configurations. The numbers of cells that have been inactivated is L1: 321, L2: 570, L3: 392, L4: 3253.

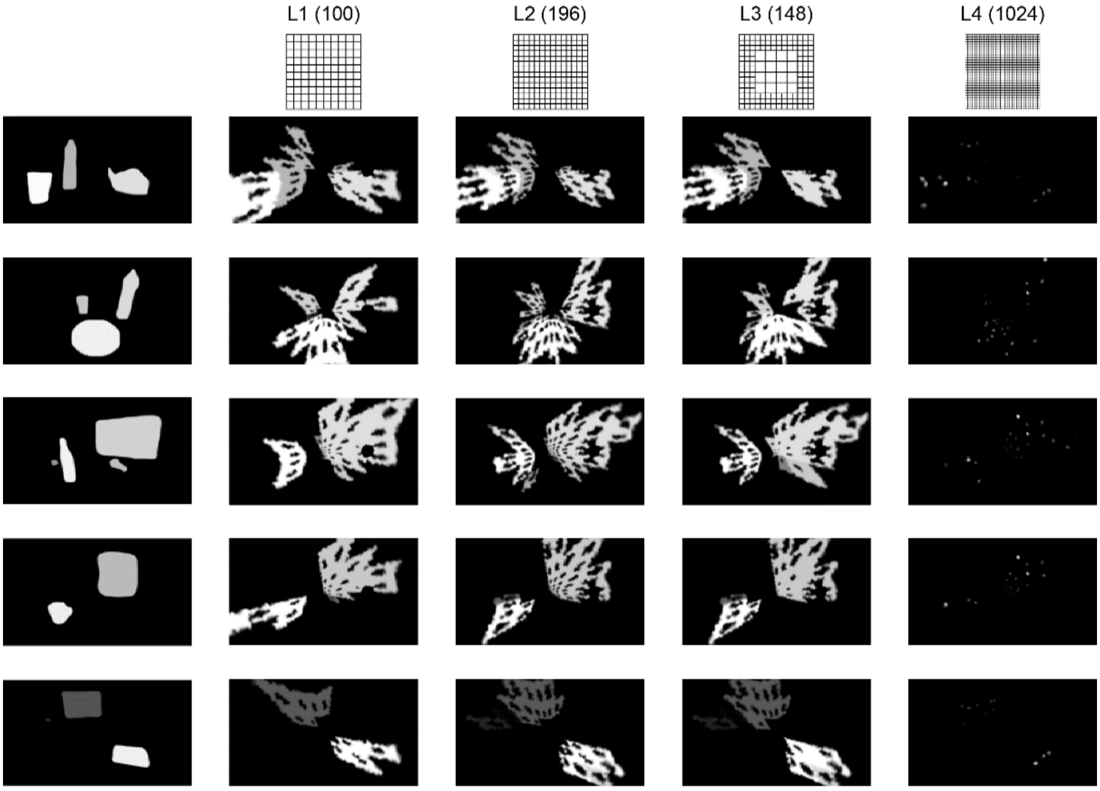

Supplementary Figure 14. ***Cell death second scenario****, related to* ***Figure 8***. Reconstructed perceptions supposing the death of the fibers closer than 100 µm from each implanted stimulating site for the different tested electrode array configurations. The numbers of cells that have been inactivated is L1: 1277, L2: 2458, L3: 1725, L4: 9825.

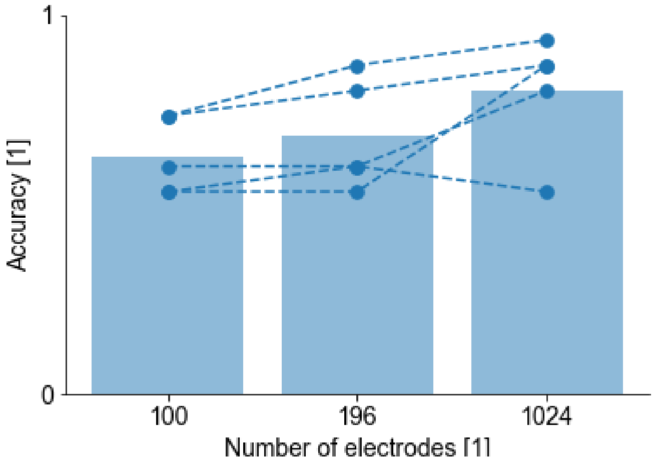

Supplementary Figure 15. ***Object recognition ability varying optic nerve electrode grid**, related to **Figure 7***. Object recognition accuracy in SPV experiments with varying numbers of electrodes placed in the optic nerve section. Each point corresponds to the object recognition accuracy of a subject on 15 reconstructed visual scenes, dashed lines join the accuracies from the same subject, bars indicate the average accuracy across subjects for a given electrode array.

<table>
<tr><td rowspan="3">True label</td><td>Table</td><td>54</td><td>6</td><td>0</td></tr>
<tr><td>Indoors</td><td>7</td><td>50</td><td>3</td></tr>
<tr><td>Outdoors</td><td>0</td><td>9</td><td>51</td></tr>
<tr><td rowspan="2"></td><td rowspan="2"></td><td>table</td><td>indoors</td><td>outdoors</td></tr>
<tr><td colspan="3">Predicted label</td></tr>
</table>

Supplementary Table 1. Confusion matrix resulting from the classification of the images into table, indoor and outdoor scenes. *Related to **Figure 4***.

	AE UND	AE ONS	AE RS	RE UND	RE ONS	RE RS
**Subj 1**	(0, 0, 0)	(0, 0, 1.5)	(0, 0, 0)	(0, 0, 0)	(0, 0, 0.5)	(0, 0, 0)
**Subj 2**	(0, 0, 0)	(0, 0, 1)	(0, 0, 0.5)	(0, 0, 0)	(0, 0, 0.23)	(0, 0, 0.17)
**Subj 3**	(0, 0, 0)	(0, 0, 0)	(1, 1, 1)	(0, 0, 0)	(0, 0, 0)	(0.25, 0.5, 0.5)
**Subj 4**	(0, 0, 0)	(0, 1, 2)	(0, 0, 1.5)	(0, 0, 0)	(0, 0.5, 0.5)	(0, 0, 0.45)
**Subj 5**	(0, 0, 0)	(0, 0, 0.5)	(0, 0, 0)	(0, 0, 0)	(0, 0, 0.08)	(0, 0, 0)
**Subj 6**	(0, 0, 0)	(0, 0, 0)	(0, 0, 1)	(0, 0, 0)	(0, 0, 0)	(0, 0, 0.23)
**Subj 7**	(0, 0, 0)	(0, 0, 1)	(0, 0, 1)	(0, 0, 0)	(0, 0, 0.5)	(0, 0, 0.33)
**Subj 8**	(0, 0, 0)	(0, 0, 0)	(0, 0, 0.5)	(0, 0, 0)	(0, 0, 0)	(0, 0, 0.07)
**Subj 9**	(0, 0, 0)	(0, 1, 1)	(1, 1, 2)	(0, 0, 0)	(0, 0.25, 0.5)	(0.29, 0.5, 1)
**Subj 10**	(0, 0, 0)	(0, 1, 2)	(1, 1, 2)	(0, 0, 0)	(0, 0.33, 0.5)	(0.29, 0.43, 0.5)

Supplementary Table 2. First, second and third quartiles of subject-wise distributions of absolute and relative errors for SPV experiment, task 1. *Related to **Figure 7***.

	DIS UND	DIS ONS	DIS RS	SIZ UND	SIZ ONS	SIZ RS
**Subj 1**	(1, 1, 1)	(0.43, 0.85, 1)	(0.82, 1, 1)	(0.86, 1, 1)	(-1, -0.82, 0.33)	(1, 1, 1)
**Subj 2**	(0.33, 0.91, 1)	(0.91, 1, 1)	(1, 1, 1)	(0.33, 0.91, 1)	(0.33, 1, 1)	(-0.33, 0.33, 0.83)
**Subj 3**	(0.89, 1, 1)	(1, 1, 1)	(0, 0, 0)	(0, 0.67, 1)	(0.33, 0.64, 1)	(0, 0, 0)
**Subj 4**	(0.43, 1, 1)	(1, 1, 1)	(0.83, 1, 1)	(-1, 1, 1)	(1, 1, 1)	(0.3, 1, 1)
**Subj 5**	(1, 1, 1)	(0.82, 1, 1)	(1, 1, 1)	(0.60, 1, 1)	(0.57, 1, 1)	(1, 1, 1)
**Subj 6**	(1, 1, 1)	(0.93, 1, 1)	(0.78, 1, 1)	(0.42, 0.67, 1)	(0.33, 1, 1)	(0.33, 1, 1)
**Subj 7**	(0.82, 1, 1)	(0.93, 1, 1)	(1, 1, 1)	(0.82, 1, 1)	(0.96, 1, 1)	(0.36, 0.91, 1)
**Subj 8**	(1, 1, 1)	(1, 1, 1)	(1, 1, 1)	(0.33, 1, 1)	(1, 1, 1)	(-0.5, -0.33, 1)
**Subj 9**	(1, 1, 1)	(0.61, 0.85, 1)	(0, 0, 0)	(-0.33, 1, 1)	(-0.55, -0.33, 1)	(1, 1, 1)
**Subj 10**	(1, 1, 1)	(1, 1, 1)	(1, 1, 1)	(0.73, 1, 1)	(1, 1, 1)	(1, 1, 1)

Supplementary Table 3. First, second and third quartiles of subject-wise distributions of Kendall's taus for object distance and size for SPV experiment, task 1. *Related to **Figure 7***.

	AE UND	AE ONS	AE RS	RE UND	RE ONS	RE RS
**Subj 1**	(0, 0.5, 1)	(0, 1, 1)	(1, 1, 2)	(0, 0.13, 0.33)	(0, 0.29, 0.46)	(0.5, 0.5, 0.73)
**Subj 2**	(0, 0.5, 1)	(0, 0, 1)	(0.25, 1, 1.75)	(0, 0.13, 0.33)	(0, 0, 0.31)	(0.08, 0.88, 1)
**Subj 3**	(0, 0.5, 1)	(0, 0, 0)	(1, 2, 2)	(0, 0.13, 0.33)	(0, 0, 0)	(0.5, 0.67, 0.73)
**Subj 4**	(0, 0, 0.75)	(0, 1, 1)	(1, 1, 2)	(0, 0, 0.19)	(0, 0.29, 0.5)	(0.5, 0.5, 0.67)
**Subj 5**	(0, 0, 0)	(0, 0, 1)	(1, 2, 2)	(0, 0, 0)	(0, 0, 0.31)	(0.5, 0.67, 0.94)
**Subj 6**	(0, 0, 0)	(0, 1, 1)	(1, 1.5, 2)	(0, 0, 0)	(0.5, 0.71, 1)	(0, 0.33, 0.5)
**Subj 7**	(0, 1, 1)	(0, 1, 1)	(0.25, 1, 2)	(0, 0.29, 0.5)	(0, 0.29, 0.33)	(0.13, 0.5, 0.5)
**Subj 8**	(0, 0.5, 1)	(0, 0.5, 1)	(1, 1.5, 2)	(0, 0.13, 0.46)	(0, 0.17, 0.5)	(0.5, 0.58, 0.92)
**Subj 9**	(0, 0.5, 1)	(1, 1, 1.75)	(0.25, 1, 1)	(0, 0.13, 0.31)	(0.38, 0.5, 0.63)	(0.06, 0.38, 0.63)
**Subj 10**	(0, 0, 0.75)	(0, 1, 1)	(1, 2, 2)	(0, 0, 0.19)	(0, 0.42, 0.5)	(0.5, 0.58, 0.92)

Supplementary Table 4. First, second and third quartiles of subject-wise distributions of absolute and relative errors for SPV experiment, task 3. *Related to **Figure 7***.
